# DNA vaccines as promising immuno-therapeutics against cancer: a new insight

**DOI:** 10.3389/fimmu.2024.1498431

**Published:** 2025-01-13

**Authors:** Alireza Shariati, Arya Khezrpour, Fatemeh Shariati, Hamed Afkhami, Aref Yarahmadi, Sajad Alavimanesh, Sina Kamrani, Mohammad Hossein Modarressi, Pouria Khani

**Affiliations:** ^1^ School of Medicine, Tehran University of Medical Sciences (TUMS), Tehran, Iran; ^2^ Department of Genetics, North Tehran Branch, Islamic Azad University, Tehran, Iran; ^3^ Cellular and Molecular Research Center, Qom University of Medical Sciences, Qom, Iran; ^4^ Nervous System Stem Cells Research Center, Semnan University of Medical Sciences, Semnan, Iran; ^5^ Department of Medical Microbiology, Faculty of Medicine, Shahed University, Tehran, Iran; ^6^ Department of Biology, Khorramabad Branch, Islamic Azad University, Khorramabad, Iran; ^7^ Student Research Committee, Shahrekord University of Medical Sciences, Shahrekord, Iran; ^8^ Cellular and Molecular Research Center, Basic Health Sciences Institute, Shahrekord University of Medical Sciences, Shahrekord, Iran; ^9^ Department of Orthopedic, Faculty of Medicine, Guilan University of Medical Sciences, Rasht, Iran; ^10^ Department of Medical Genetics, School of Medicine, Tehran University of Medical Sciences (TUMS), Tehran, Iran

**Keywords:** DNA vaccines, cancer, immunotherapy, immune response, vaccines

## Abstract

Cancer is one of the leading causes of mortality around the world and most of our conventional treatments are not efficient enough to combat this deadly disease. Harnessing the power of the immune system to target cancer cells is one of the most appealing methods for cancer therapy. Nucleotide-based cancer vaccines, especially deoxyribonucleic acid (DNA) cancer vaccines are viable novel cancer treatments that have recently garnered significant attention. DNA cancer vaccines are made of plasmid molecules that encode tumor-associated or tumor-specific antigens (TAAs or TSAs), and possibly some other immunomodulatory adjuvants such as pro-inflammatory interleukins. Following the internalization of plasmids into cells, their genes are expressed and the tumor antigens are loaded on major histocompatibility molecules to be presented to T-cells. After the T-cells have been activated, they will look for tumor antigens and destroy the tumor cells upon encountering them. As with any other treatment, there are pros and cons associated with using these vaccines. They are relatively safe, usually well-tolerated, stable, easily mass-produced, cost-effective, and easily stored and transported. They can induce a systemic immune response effective on both the primary tumor and metastases. The main disadvantage of DNA vaccines is their poor immunogenicity. Several approaches including structural modification, combination therapy with conventional and novel cancer treatments (such as chemotherapy, radiotherapy, and immune checkpoint blockade (ICB)), and the incorporation of adjuvants into the plasmid structure have been studied to enhance the vaccine’s immunogenicity and improve the clinical outcome of cancer patients. In this review, we will discuss some of the most promising optimization strategies and examine some of the important trials regarding these vaccines.

## 1 Introduction

It can be argued that one of humankind’s most prominent medical struggles in the 21^st^ century is to decrease the rate of mortality and morbidity inflicted by malignancies. Based on a paper by the American Cancer Society, 1,958,310 new cancer cases and 609,820 cancer deaths are projected to occur in the United States in 2023 (1). Statistics like this indicate the necessity of developing and deploying new and more effective cancer therapeutics. Until now, the most effective cancer therapies are the conventional ones, like surgery, chemotherapy, and radiotherapy. Surgery is mostly restricted to low-grade cancers that have not metastasized. Chemotherapy, although potent enough to induce and maintain remission in many early-stage cancers, takes its toll on the patient by causing numerous side effects including nausea, fatigue, hair loss, and neuropathies, some of which can have significant adverse effects on the psychosocial and physiological states of the patient, which in turn worsen the prognosis. This consequence stems from the fact that chemotherapy does not distinguish between normal and cancerous cells and poisons them all, resulting in the malfunction of many normal systems. Radiotherapy has its side effects as well, mostly dependent on the body part being irradiated, including headache, hair loss, diarrhea (a symptom of radiation colitis), and urologic complications to name a few (2–4). It should be mentioned that novel technology to reduce the exposure of normal tissue during radiotherapy such as stereotactic body radiotherapy (SBRT) has significantly diminished the occurrence and intensity of the side effects previously mentioned (5). Apart from these acute and subacute side effects, chemotherapy and radiotherapy have been linked with some late-onset complications, the most important of which are secondary malignancies caused by these therapeutic agents. The malignancies most commonly associated with chemotherapy and radiotherapy are of hematogenous origin, namely myelodysplastic syndrome (MDS) and acute myeloid leukemia (AML). Higher dose intensities and longer treatment periods increase the risk of acquiring these secondary cancers (2, 3). Apart from the side effects, these conventional treatments are not potent enough to fight off some types of cancer. For example, despite all of the innovations in chemotherapeutic regimens and surgical techniques, the combined stage-independent five-year survival rate of patients with pancreatic cancer is below 10 percent. This rate falls to around 1 percent in patients with stage IV pancreatic cancer (6, 7). These numbers show that our current arsenal to fight some types of cancer is simply insufficient and we need to expand our therapeutic options with more efficacious modalities to be able to improve our patients’ outcomes. Novel therapeutics can change the landscape of cancer management. These therapeutics mostly focus on activating the immune system to detect and destroy tumor cells selectively. Tumor cells have developed strategies to create a hostile micro-environment for the immune system to avoid recognition by it. Antigen loss, secretion of immunosuppressive cytokines, and downregulation of MHC molecules are just a few examples of these strategies. By manipulating the immune system to target tumor antigens, novel cancer treatments harness the selectively exerted power of the immune system and redirect it against the growing tumor. It should be obvious that the involvement of the immune system in this process means there are fewer off-target side effects associated with these treatments than conventional treatments such as chemotherapy. It should be mentioned that it is not our objective to convey that novel cancer therapeutics should replace traditional treatments, rather a combination of both of these modalities will most probably result in the optimal immunological and clinical responses for cancer patients (8, 9).

These innovative therapeutics include immune checkpoint blockers (ICBs), Chimeric Antigen Receptor T-cells (CART-cells), and cancer vaccines (10, 11).

All vaccinations have the primary objective of preventing illnesses and their potentially fatal effects (12). Any particular vaccination functions by stimulating the body’s defenses against an antigen present in the intended infection (13). Gene vaccination is the most notable vaccine technique of the 21st century. Utilizing developments in biochemistry, molecular biology, genetics, and chemistry, gene vaccines transfer specific segments of the target pathogen’s messenger ribonucleic acid (mRNA), or deoxyribonucleic acid (DNA). Theoretically, engineering might eradicate all pathogenicity, which would minimize vaccination morbidity and prevent vaccine-related deaths due to inoculum pathogenesis (14, 15). Cancer vaccines are designed to deliver tumor antigens to antigen-presenting cells (APCs). In turn, these cells present the antigens to T-cells to activate both cellular and humoral immune responses (16, 17).

The purpose of this study is to focus on a specific subgroup of nucleotide-based cancer vaccines, the DNA cancer vaccine.

## 2 DNA cancer vaccines

**Figure 1:**
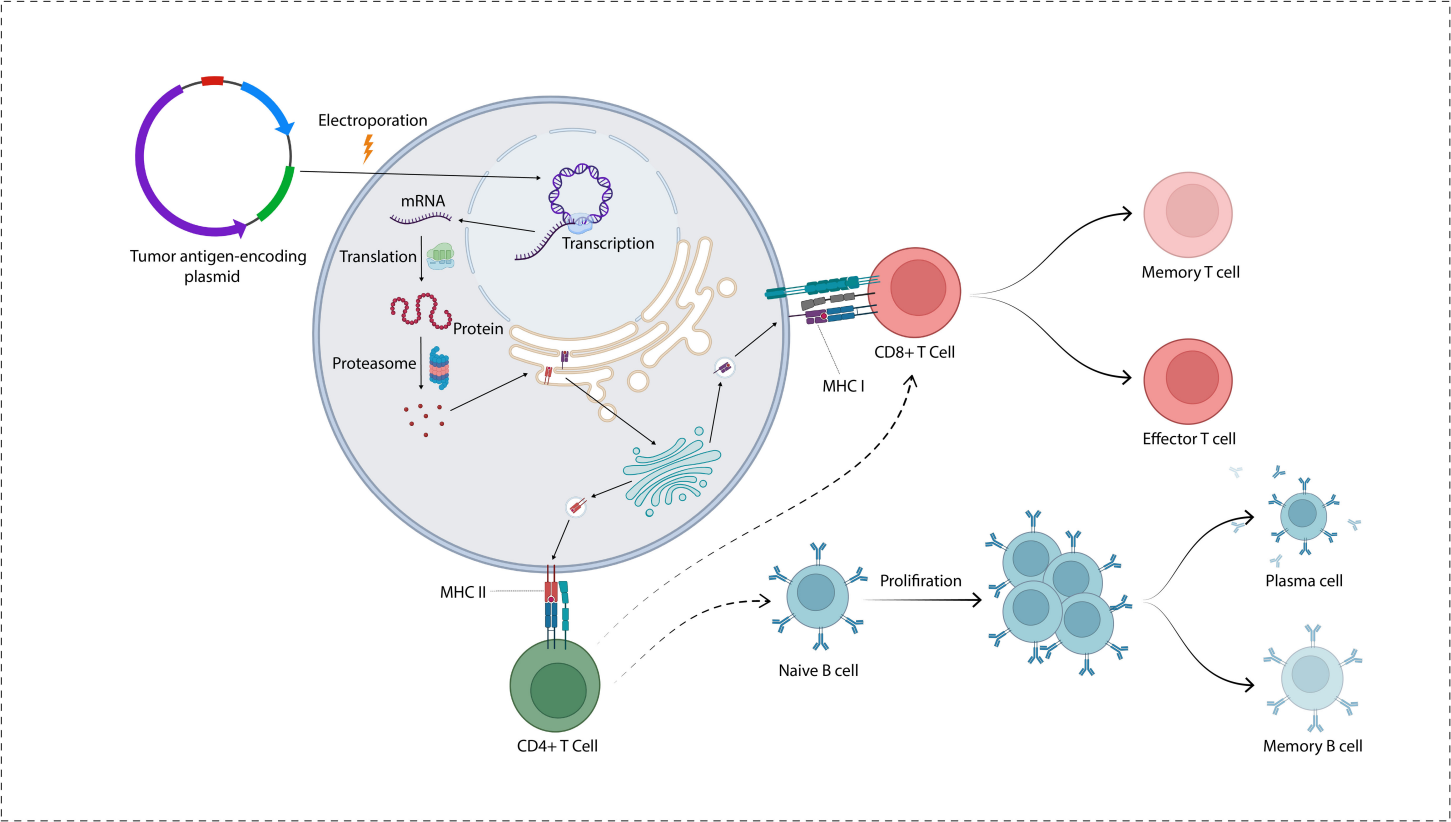
A plasmid encoding tumor antigens is transported into the cytoplasm and cell nucleus. Following transcription, the proteins go through degradation by the action of proteasome. The antigenic peptides are transported into the endoplasmic reticulum where they are loaded on MHC molecules to be presented on the cell surface.

**Figure 2:**
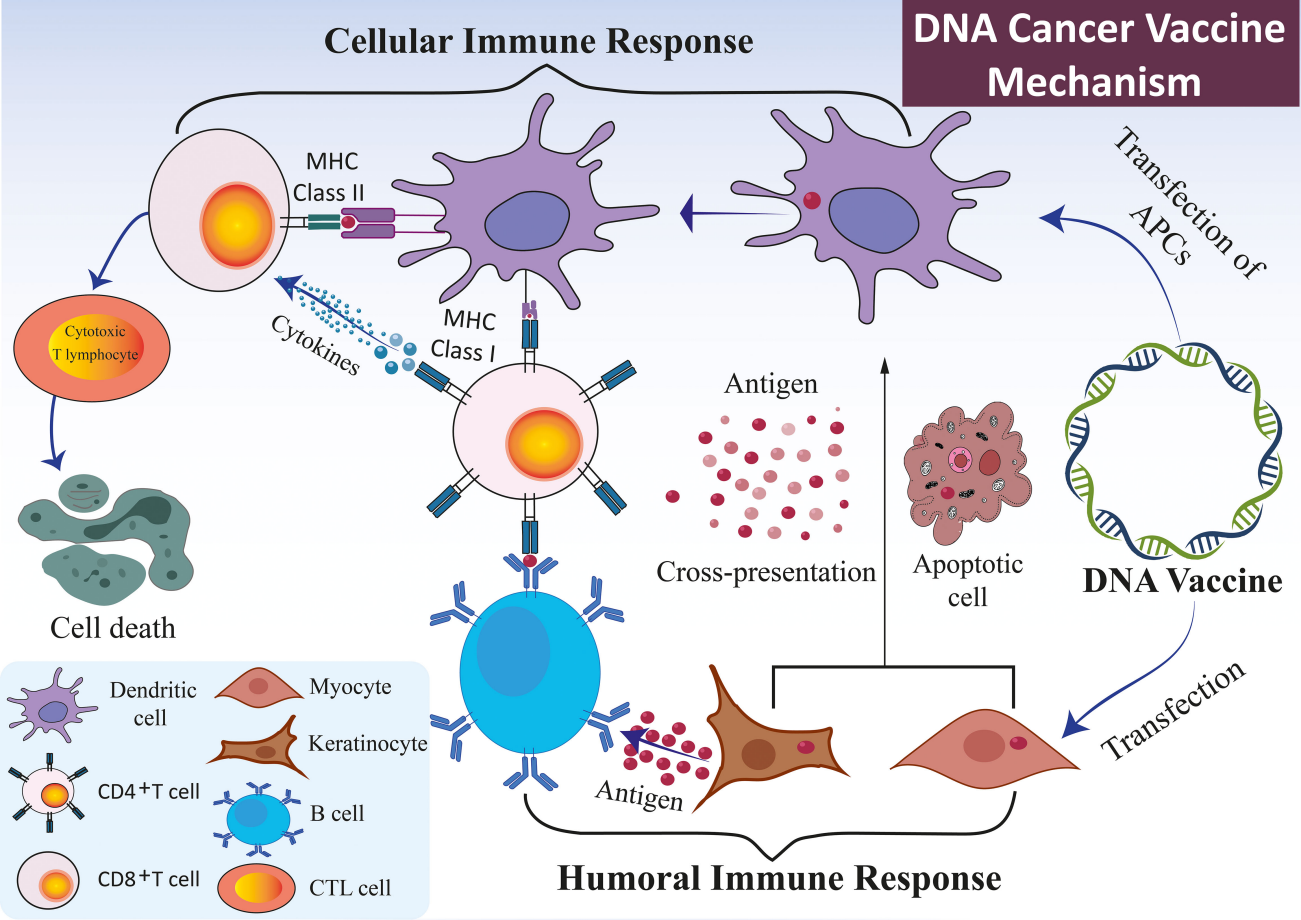
Mechanism of DNA vaccines. The immune response elicited by DNA vaccines, which encode specific antigens, primarily occurs through the direct transfection of APCs, such as DCs, keratinocytes, and myocytes. DNA vaccines administered to keratinocytes or myocytes facilitate the expression of antigen genes via exosomes or apoptotic bodies. These structures release derived peptides and proteins, subsequently internalized by DCs. The DCs preferentially present the antigens to CD4+ T cells through MHC II, eliciting a cellular immune response. An alternative pathway involves the direct transfection of APCs, which results in the expression of endogenous antigen genes. This expression occurs concurrently through MHC I and MHC II molecules, thereby simultaneously stimulating both CD8+ and CD4+ T cell responses. In conjunction with the cellular immune response, the B cell receptor identifies protein antigens derived from somatic cells, such as myocytes or keratinocytes. This recognition is facilitated by the assistance of pre-activated, antigen-specific CD4+ T cells capable of initiating a humoral immune response.

Evidence indicating that mammalian cells can express foreign plasmid DNA following transfection, compelled researchers to use DNA as a vehicle for transduction of peptide blueprints. This principle can be useful in treating many disorders including allergies, autoimmune disorders, genetic disorders, and malignancies. The recent progress in recombinant DNA technology paved the way for the development of DNA vaccines, such that the plasmid DNA can contain any desired combination of genetic information; from viral genes to various cancer genes (18). The advances in whole-genome sequencing have enabled us to analyze the entire genome of tumor cells and detect thousands of TSAs, the results of which can be utilized to tailor cancer treatment for each patient. Furthermore, transcriptomic studies (e.g. DNA microarrays) have enabled us to determine the degree of expression of different genes in cancer cells and find TAAs. These antigens can then be incorporated into a plasmid and delivered to the patient to stimulate an anti-tumor immune response. This combination of target acquisition and the relative ease of plasmid design and manufacturing has made this methodology appealing and subject to many clinical trials. DNA cancer vaccines are composed of plasmid DNA molecules that encode TAAs or TSAs associated with the targeted tumor under the control of a mammalian promoter (19). After the transfection of the host cell and transportation of the plasmid into the nucleus of the transfected cell, TAAs or TSAs are expressed by the host’s molecular machinery. After processing, immunogenic epitopes are loaded on the MHC proteins and will ultimately activate the CD4+ and CD8+ T-cells and their associated cellular and humoral immune response. DNA vaccines can be administered through several routes, the most prominent ones being the intradermal and intramuscular. In either case, if APCs are directly transfected, they will primarily present the endogenous antigen to the CD8+ cells through MHC-I, although a portion of the antigens are cross-presented through MHC-II to CD4+ T-cells which mostly activate the humoral immune response (**Figures 1**, **2**) (20, 21). Myocytes and keratinocytes can also pick up the plasmid and express the tumor antigen. Upon phagocytosis by APCs, the antigen is processed and presented to the CD4+ T-cells on MHC-II. Although myocytes are efficient in expressing the tumor antigens, APCs are specialized immune cells for processing and presenting such antigens to the adaptive immune system and hence, are pivotal to this method of cancer vaccination (22–24). The activated immune cells can potentially recognize the tumor cells and destroy them through different mechanisms including the induction of apoptosis and phagocytosis. The main hurdle in DNA vaccines’ application is their relatively poor immunogenicity. Cancer cells utilize many different strategies to evade the immune system. To overcome this challenge, researchers are developing new tricks to strip cancer cells from their defense mechanisms. For instance, the incorporation of immune-stimulatory cytokines such as GM-CSF into plasmids has been shown to improve immunological and clinical responses. Many other strategies to improve DNA cancer vaccines are being evaluated, some of which will be discussed later (25).

Nucleotide-based vaccines such as DNA vaccines offer several advantages over peptide vaccines. DNA vaccines can encode several full-length antigens, whereas peptide vaccines usually induce an immune response against a single epitope. DNA vaccines can also incorporate immune-modulatory molecules which augment the resulting immune response. They are also relatively easier to produce with fewer systemic side effects (26).

Compared to mRNA cancer vaccines, there are some advantages and disadvantages associated with DNA cancer vaccines. DNA vaccines are more easily diffused across membranes, making them more suitable for lipid-based delivery (27, 28). Also, DNA molecules have a longer half-life compared to mRNA molecules which allows for a more prolonged immune response (28). However, the production process of mRNA vaccines is simpler and cell-free (29, 30). Also, DNA vaccines may induce insertional mutagenesis which can result in catastrophic outcomes. Since the production process of DNA vaccines is not usually cell-free, the injection can result in the entry of contaminating microorganisms which can potentially cause serious infections in the immunocompromised cancer patient (27, 31).

## 3 Advantages and disadvantages

DNA vaccines offer several advantages over conventional treatments and other modalities of cancer vaccination. Compared to conventional methods of cancer treatment, like chemotherapy and radiotherapy, they are far less associated with systemic side effects, giving rise to a systemic immune response that is as effective on metastatic lesions as on the primary tumor. They also induce a memory immune response that will lower the incidence of recurrence (32, 33). DNA vaccines can be easily mass-produced, they are stable (unlike mRNA vaccines that usually require ultra-low storage temperature), cost-effective, and easily stored and transported. They can be tailored to each patient, personalizing the treatment. They are relatively safe, usually well-tolerated, and multiple administrations are associated with no significant side effects although mild autoimmune reactions have been reported. Unlike viral vaccines, they are not associated with pathogenic infection (34–37), and multiple administrations of these vaccines are not associated with the production of neutralizing antibodies that hinder future vaccination, so multiple doses can be administered easily. Compared to mRNA cancer vaccines, DNA vaccines confer a longer production of tumor antigens which prolong the effect of the immune response, potentially decreasing the required number of vaccine doses.

One of the most important advantages of DNA cancer vaccines is the possibility of flexible design of plasmids with multiple tumor antigens and incorporation of immunomodulatory genes (such as interleukins including IL-1 or GM-CSF and Toll-like receptor (TLR) agonists) (18). The innate ability of bacterial plasmid to stimulate the immune system can also be exploited in this method as several DNA sensing molecules in the cytoplasm of immune cells are activated following the plasmid transfection which will in turn augment the activation of the innate immune system (38). The incorporation of CpG islands and double-stranded DNA structures can enhance the activation of the innate immune system.

Clinical trials almost unanimously point to the activation of both humoral (facilitated by CD4+ T-cells) and cellular immune responses (mostly by the activation of CD8+ T-cells) following DNA vaccination which means many different mechanisms work synergistically to eliminate the tumor.

The major disadvantage of DNA cancer vaccines which is limiting their application in clinical settings is their poor immunogenicity; Several factors contribute to this inefficiency. Firstly, cells do not readily pick up the plasmid; Various methods such as electroporation and sonoporation are used to circumvent this issue. Electroporation uses small electric chargers to transiently permeabilize the cellular membrane and allow for the passage of plasmids into cells (39–43). Also, the encoding DNA does not readily spread from cell to cell *in vivo* which limits the number of immune cells activated by each plasmid. Secondly, because TAAs are self-antigens, central and peripheral mechanisms of tolerance, limit their immunogenicity (44); Combination therapy with ICB is a suitable candidate for overcoming this problem, albeit slightly increasing the risk of autoimmunity (45–49). Also exploiting TSAs instead of TAAs can help us go around the problem of tolerance, as TSAs are not subject to central and peripheral tolerance. Another major obstacle in the development of all cancer vaccines is tumor resistance. As will be described later, a large part of the struggles to improve these vaccines rely on solving the resistance problem. Cancer cells can develop resistance to these vaccines in two major ways:

- Tumor intrinsic resistance: Loss or mutation of vaccine-targeted epitopes, altered antigen processing pathway, and loss of HLA expression (50).
- Tumor extrinsic resistance: Immunosuppression in the TME like infiltration of Tumor-associated macrophages (TAMs), regulatory T-cells, and myeloid-derived suppressor cells (MDSCs) which dampen the immune response and cause T-cell exhaustion. TAMs are specifically capable of secreting multiple immuno-inhibitory cytokines such as IL-10 and TGF-B (51–53).

There are several methods currently being investigated as means to increase the immunogenicity of DNA cancer vaccines which could turn immunologically “cold” tumors into “hot” ones. Another issue is the high production time required for personal vaccine development, a time most cancer patients do not have. Most of this time is spent on finding and prioritizing tumor antigens (18). What can we do about it?

Also, as with any other DNA fragment, there is a risk of insertional mutagenesis, albeit this risk is too low to outweigh the benefits of DNA vaccines. Moreover, these vaccines are limited to inducing immune responses against peptide antigens as they cannot encode non-peptide antigens like saccharides. Also, there is a slight chance of horizontal transfer of antibiotic resistance genes from the vaccine plasmids into gut microbiota which could complicate the treatment of GI infections in treated patients (54).

## 4 Optimization strategies

Several strategies have been proposed for DNA vaccine optimization. These can be categorized into four major classes:

1. Structural modification
2. Delivery methods
3. Combination therapy
4. Route of administration

We will discuss each class in more detail.

## 5 Structural modification

One of the most promising avenues to increase DNA cancer vaccine efficiency is to optimize its structure. To do this we can manipulate the sequence of coding and non-coding regions of the plasmid. In this section, we are going to explore more about these techniques.

Early in the course of vaccine development, it was realized that the first important aspect of making a potent vaccine is choosing the right antigens. Thousands of TAAs and TSAs can be detected in a tumor specimen. Apart from finding these antigens, one needs to prioritize them based on their level of immunogenicity. Several in-silico methods and computer programs are developed and being developed to improve the prediction of antigen immunogenicity considering different factors including the binding affinity to MHC molecules (55, 56). Artificial intelligence is another upcoming tool that can be used for the prediction of antigen immunogenicity antigen-MHC interaction affinity. Tools such as NetMHC and NetMHCpan can be quite useful in this field (57). By helping with the antigen selection and modification process, these AI tools might have an invaluable effect on optimizing DNA cancer vaccines.

Also, the nuclear import of plasmids is a critical step toward tumor-antigen expression. Incorporation of nuclear localization sequence (NLS) into the plasmid can help facilitate the transportation of plasmids into the nucleus through nuclear pore complexes (NPCs) (58).

To develop a potent immune response, the expression of the antigens encoded on the plasmid should be optimized. One study indicated that a more powerful promoter induces a more potent immune response by producing more antigens (59). Strong promoters such as those of CMV and SV40 are usually preferred over eukaryotic promoters as endogenous promoters are not as potent as viral ones (58). Yet these viral promoters have their downsides as well, the most important one being their inhibition in inflammatory conditions as a direct act of pro-inflammatory cytokines (60).

Using codon optimization over wild-type codons is another hot topic in improving DNA vaccines. Codons can be optimized in many different ways. Codons with more abundant cognate tRNA, with a higher ratio of guanine and cytosine, more abundant in the sequence genes encoding highly-expressed proteins in humans, and not forming highly stable secondary mRNA structures such as hairpins are preferred (61, 62).

The Kozak sequence functions as a translation initiation site in most eukaryotic mRNA transcripts. Regarded as the optimum translation initiation sequence in eukaryotic cells, it has been used in DNA vaccines before the initiation codon. Using human-specific codons, poly-A tails, nucleoside modification, and optimized untranslated regions (UTRs) can also help with transcription, mRNA export from the nucleus, and mRNA stability (63). Epitope enhancement refers to the process of modifying epitopes to increase their affinity for MHC molecules by incorporating “anchor residues” which can increase binding and immunogenicity (58).

Many DNA cancer vaccines also possess unmethylated CpG islands and double-stranded DNA structures which act as PAMPs that activate the TLR9 signaling pathway of the innate immune response with subsequent release of immune-stimulatory cytokines (25); Specifically, DNA sensors like DAI, IFI16, H2B, and LRRFIP1 will activate Tank-binding kinase1 (TBK1) and STING pathway, leading to activation of IRF3 and production of Type 1 IFN (38). Also, CRISPR/Cas9 technology, as a DNA-editing tool can be used to optimize the sequence of the antigen-encoding plasmid. This can be achieved in many ways such as codon and promoter optimization (64). Further investigation into this application and its combination with AI tools can have a valuable impact on the optimization of DNA cancer vaccines.

A strategy to overcome immune tolerance associated with using TAAs in DNA cancer vaccines is to utilize xenogeneic antigens. These antigens are phylogenetically conserved proteins that are capable of evoking an immune response against the native TAAs. Because of the sequence homology between these antigens and their human counterparts, xenogeneic antigens bypass the immune tolerance. In other words, they are different enough from self-antigens to break the tolerance, yet similar enough to elicit an effective antigen-specific immune response (60, 65). ONCEPT is a vaccine against canine oral melanoma, among other off-label targets, composed of plasmid DNA encoding human tyrosinase, an enzyme highly conserved among the mammalian species which is crucial for the melanin synthesis pathway. The vaccine is delivered via VetJet, an FDA-approved needleless transdermal delivery device. Four biweekly intradermal injections are administered and a booster dose is applied every six months in case of survival. The first trial evaluating the clinical efficacy of this vaccine demonstrated a significant increase in the median survival time in dogs receiving the vaccine compared to historical controls, although there are some controversies regarding the validity of the results (66). In a phase I/II study conducted by Seledtsov and coworkers, 40 patients with stage III/IV malignant melanoma were treated with a poly-epitopic xenogeneic DNA cancer vaccine prepared from murine B16 and LLC murine melanoma cell lysates, combined with subcutaneous recombinant IL-2 injection. Delayed-type hypersensitivity reaction (DTH) showed a remarkable level of immune-reactivity, especially toward B16 melanoma antigens. Compared to historical controls, a sizably longer median overall survival was observed among xeno-vaccinated patients. Interestingly, patients with stronger DTH reactivity had better clinical outcomes (67, 68).

Since few studies have found the immune response against DNA vaccines encoding xenogeneic antigens to be robust enough to significantly alter the clinical course of cancer patients, the attention shifted toward vaccines encoding chimeric antigens. By combining elements of both xenogeneic and autologous antigens, these hybrid vaccines ensure bypassing tolerance and induction of a potent immune response (60). One such vaccine targeted HER-2 receptors on HER2+ breast and pancreatic cancer cells. HER-2 or ERBB2 is the human epidermal growth factor receptor 2 which is a membrane-bound receptor tyrosine kinase, acting as a proto-oncogene in some tumors including breast, ovarian, and lung cancer. Following binding to a ligand, heterodimerization results in autophosphorylation and subsequent activation of several intracellular signaling pathways including mitogen-activated protein kinase (MAPK). A group of breast cancer cells over-express this protein by a mechanism known as amplification, which results in the expansion of this gene’s copy number. Also, structural mutations can result in ligand-independent constitutive dimerization and uncontrollable cellular growth (68, 69). A DNA vaccine containing DCs loaded with plasmids encoding chimeric rat/human HER2 was able to induce T-cell mediated immunity in patients with pancreatic and HER2+ breast cancer with confinement of tumor growth and overcoming the suppressor effects of regulatory T-cells (T_regs_), IL-10, and TGF-β (70).

Also, the fusion of self-ortholog with bacterial toxins has been shown to improve the weak immunogenicity of self-antigens by utilizing a concept called associative recognition. For instance, the epitopes of the tetanus toxin (TT) are highly immunogenic to both innate and adaptive immune cells. By fusing a DNA fragment encoding TT to the DNA cancer vaccine, an intense immune response mediated by CD4+ and CD8+ T-cells can be induced against the self-antigen which can, in turn, improve the clinical profile of cancer patients (71–73). In a phase I/II study, patients with prostate cancer received a DNA cancer vaccine encoding a chimeric protein comprising a domain of tetanus toxin fragment C and HLA-A2^+^-binding prostate membrane-specific antigen (PMSA). All 32 patients were HLA-A2^+^. The vaccine was injected intramuscularly with or without electroporation. No objective decline of serum PSA was observed but a significant deceleration of disease progression was noted among vaccinated patients compared to controls. A minor increase in clinical and immunological response was recorded among those receiving electroporation after vaccination (74).

A more personalized and effective approach to cancer treatment would be targeting tumor-specific antigens or neo-antigens which are the products of non-synonymous somatic mutations. They offer several advantages over TAAs. Firstly, they are tumor-specific, meaning that they are not amenable to immune tolerance, making them significantly more immunogenic. Secondly, there are fewer side effects associated with utilizing these antigens because they are not present in normal tissues. Lastly, they are quite useful in many solid tumors because of the scarcity of immunogenic TAAs in these tumors (18, 75–81). Some clinical trials have shown that delivering neo-antigens via DNA vaccines induces a more robust immune response compared to delivery with mRNA vaccines and peptide vaccines (82). Using neo-antigens in mRNA vaccines mediated the upregulation of PD-1 and PDL-1 in the TME. Thus the application of ICBs blocking these molecules is extended in this mode of treatment. To design these vaccines, firstly, tumor mutations should be detected. After tumor biopsy and whole-exome sequencing, non-synonymous mutations are detected through comparison with whole-exome sequencing of non-cancerous cells of the affected individual. Then TSAs are ranked based on their immunogenicity using in silico prediction algorithms and *in vivo* binding assays (83–85). For these vaccines to be applicable, especially for patients with advanced metastatic disease, the manufacturing time shall be shortened which is currently about 4.5 months from discovery to production (86). Another thing that should be considered is that cancer cells show striking genetic heterogeneity and usually, a single TSA cannot elicit an immune response that targets all cancer cells. A way to overcome this problem is by incorporating multiple TSAs into one plasmid, augmenting and broadening the immune response to target each cancer cell at least once (82, 87, 88). In a phase I single-arm open-label clinical trial, the safety and efficacy of a DNA cancer vaccine encoding a fusion gene of patient-specific single chain variable fragment (also known as Idiotype) linked to fragment C of tetanus toxin was evaluated in multiple myeloma patients with complete remission (CR) or partial remission (PR) following chemotherapy and autologous bone marrow stem-cell transplant. Id-specific T-cell response was detectable in 21 percent of patients. Over 52 weeks of study, CR/PR was maintained in 79 percent of the patients and the median time to progression was 38 months. The overall survival was about 64 percent after 86 months of follow-up (89). In a phase 1 clinical trial, patients with metastatic hormone-sensitive prostate cancer (mHSPC) received a DNA vaccine encoding TSAs combined with ipilimumab (anti-CTLA4), nivolumab (anti-PD1), and Prostvac. By utilizing both DNA vaccination and Prostvac, both TAAs and TSAs are targeted which can broaden the scope of the immune response. The DNA vaccine is injected intramuscularly followed by electroporation. Although the trial is marked as completed in clinicaltrials.gov, we were not able to find its results (NCT03532217). In a phase I clinical trial in the recruiting stage, patients with MGMT promoter unmethylated glioblastoma will receive a personalized neoantigen DNA vaccine intramuscularly with electroporation, combined with retifanlimab (anti-PD1 monoclonal antibody). Each patient will receive 3-6 doses of the vaccine. The primary objective is to assess the safety and tolerability of the vaccine. The secondary objective is to evaluate the level of immunogenicity determined by T-cell reactivity to neoantigens, T-cell receptor (TCR) sequencing to assess T-cell clonality, flow cytometry, and measurement of pro- and anti-inflammatory chemokines and cytokines as assessed by multiplex ELISA (NCT05743595).

Also, the incorporation of multiple cancer antigens into a plasmid, could strengthen the vaccine and broaden the resulting CTL response. Some tumor cell clones can silence, delete, or mutate the vaccine-targeted antigens; Using poly-epitope vaccination, one can ensure a full-scale immunologic ambush of the tumor. This method could also overcome problems such as the absence of appropriate T-cell repertoire or variances in the MHC haplotype (90). Interestingly, as Palmowski et al. demonstrated, it has been observed that even with poly-epitope cancer vaccines, usually, the immune response is far more potent against one of the epitopes; That epitope is said to have immunodominance (91, 92). The immunodominance relies mostly on the affinity of the selected epitopes for MHC molecules and transporters (56). In a phase 1 clinical trial, a single poly-epitope DNA vaccine was injected in 18 patients with triple-negative breast cancer who had persistent disease following neoadjuvant chemotherapy. The vaccine was well-tolerated and the adverse events were restricted. Using flow-cytometry, the neo-antigen-specific immune response was noted in 16/18 subjects, and high extension of T cell receptor (TCR) clonotypes was noted in all patients. Survival after 36 months in those patients who received the vaccine, was 87.5%, compared to 49% in historical control patients (93). In a phase I clinical trial in the recruiting stage, a DNA vaccine encoding multiple tumor antigens (including CD105, Yb-1, SOX2, CDH3, MDM2) called STEMVAC, is injected intradermally together with sargramostim (GM-CSF) into patients with stage IV non-small cell lung cancer (NSCLC). The patients will receive 3 vaccine doses with one booster shot. They are projected to be followed twice a year for up to five years after the completion of the vaccination (NCT05242965).

**Figure 3:**
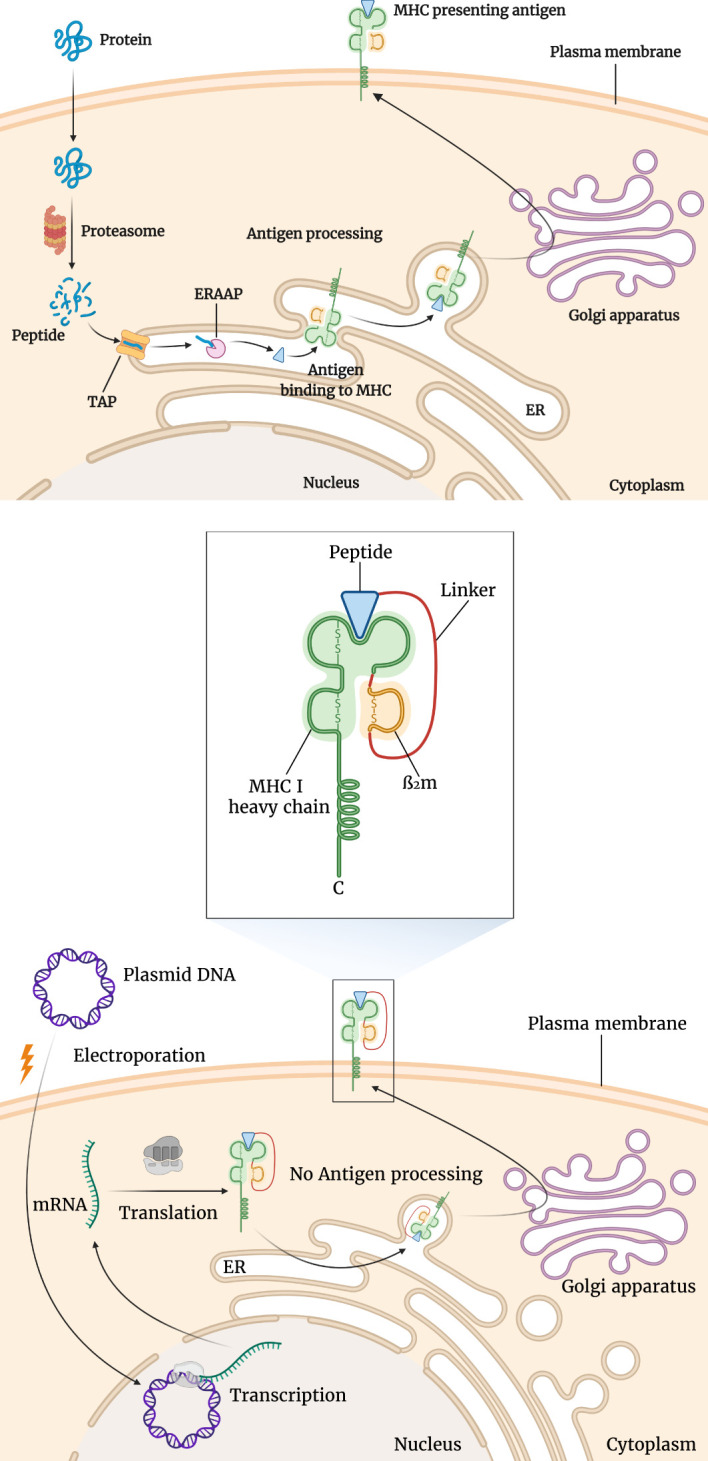
Differences of antigen presentation by conventional MHC and single chain trimer (SCT). For conventional antigen presentation, following protein splicing, the resulting short peptides are transported into the endoplasmic reticulum (ER) where MHC molecules await them. Following the binding of the peptide antigen to the MHC molecule, it is transported to the cell surface to be presented to T-cells. This is shown in the upper section of the figure. However, as seen in the lower section of the figure, using SCTs several of these steps are bypassed. SCT is a fusion protein composed of MHC I heavy chains, β2 microglobulin, and antigenic peptide. After the transfection of the encoding plasmid, the SCT is expressed and transported through the ER to the cell surface.

Another incredibly innovative technique is utilizing Single Chain Trimers (SCTs). SCTs are secreted chimeric proteins, consisting of an MHC1 heavy chain, β2 microglobulin, a peptide antigen, IgG immunoglobulin, and a linker. The major advantage of this approach is the circumvention of the antigen processing pathway that may be suboptimal (**Figure 3**). Furthermore, dimerization of the IgG portion of the protein, mediates the internalization of the protein into APCs, after its recognition by Fc receptors on these cells. In this way, antigen processing assumes an auxiliary role in provoking the immune response (94). Importantly, intradermal administration of a DNA vaccine encoding an SCT chimeric protein, containing tyrosinase-related protein 2 (Trp2), a melanoma antigen was able to elicit a powerful Trp2-specific CD8+ T cell-mediated immune response and suppress B16 tumor growth and spread in a murine model (95). In another trial, a DNA vaccine encoding an SCT, containing a VEGFR2 (vascular endothelial growth factor receptor 2) antigen peptide was evaluated on C57BL/6 mice. VEGFR2 is the most important target of anti-angiogenic therapy. Each mouse was immunized three times intradermally. A robust cell-mediated immune response against VEGFR2 was noted using an LDH (lactate dehydrogenase) assay. A significant suppression of tumor-mediated angiogenesis was observed using alginate bead analysis and IHC staining. Also, a sizable reduction of tumor metastases was observed in mice with B16 melanoma or 3LL Lewis lung carcinoma in autopsy. Overall the vaccine was successful in inducing an immune response powerful enough to suppress the VEGFR2-induced angiogenesis (96).

## 6 Adjuvants and combination strategies

Combining DNA vaccination and other immunotherapeutic and conventional therapies can augment the resulting immune response through a synergistic or additive effect. Several combinatorial approaches have been suggested, some of which will be discussed here. A summary of the clinical studies is given in **Table 1**.

**Table 1:** Clinical trials of DNA cancer vaccines extracted from clinicaltrials.gov.

NCT	Phase	Status			Indication	Formulation type	Combination	Route	Sponsor
**NCT02139267**	Phase II	Completed			Cervical intraepithelial neoplasia 3 (CIN3)	GX-188E encoding HPV E6 and E7, a tissue plasminogen activator signaling sequence, and FMS-like tyrosine kinase 3 ligand	–	IM with electroporation	Genexine, Inc
**NCT00104845**	Phase I	Completed			Stage IIB, IIC, III, and IV melanoma	Arm I: receiving human gp100-encoding DNA Arm II: receiving murine gp100-encoding DNA	–	IM	Memorial Sloan Kettering Cancer Center
**NCT02348320**	Phase I	Completed			Triple-negative breast cancer, persistent following neoadjuvant chemotherapy	Personalized naked DNA encoding multiple neo-antigens	–	IM with electroporation	Washington University School of Medicine
**NCT00199849**	Phase I	Completed			Prostate Cancer, Bladder Cancer, Non-small Cell Lung Cancer, Esophageal Cancer, Sarcoma	NY-ESO-1 encoding DNA	–	ID using XR-1 Powderject	Ludwig Institute for Cancer Research
**NCT00859729**	Phase I/II	Completed			Relapse of Prostate Cancer	Rhesus (Rh) PSA encoding DNA	–	ID with Electroporation	Uppsala University
**NCT01706458**	Phase II	Completed			Castration-Resistant, Metastatic Prostate Cancer	Sipuleucel-T with or without prostatic acid phosphatase (PAP) and rhGM-CSF encoding DNA (pTVG-HP)	–	Sipuleucel-T: IV pTVG-HP: ID	University of Wisconsin, Madison
**NCT01064375**	Phase I/II	Completed			Colorectal cancer	DNA vaccine encoding wild-type human CEA fused to a tetanus toxoid T-helper epitope (tetwtCEA)	Cyclophosphamide, GM-CSF	ID with electroporation	Maria Liljefors
**NCT01334060**	Phase II	Completed			CML, AML	DNA vaccine encoding WT1 (p.DOM-WT1)	–	IM with electroporation	University Hospital Southampton
**NCT00121173**	Phase I/II	Completed			HPV16+ Cervical Intraepithelial Neoplasia 2/3 (CIN2/3)	DNA vaccine encoding HPV16 E7 linked to Mycobacterium tuberculosis heat shock protein 70 (HSP70) and signal peptide (pNGVL4a-Sig/E7(detox)/HSP70)	–	IM	Johns Hopkins
**NCT00849121**	Phase II	Completed			Prostate cancer	pTVG-HP: DNA encoding prostatic acid phosphatase (PAP)	Rhesus GM-CSF	ID	University of Wisconsin, Madison
**NCT00680589**	Phase I	Completed			Melanoma	DNA encoding murine TYRP2	–	IM	Memorial Sloan Kettering Cancer Center
**NCT02499835**	Phase I/II	Completed			Hormone-Resistant Prostate Cancer, Metastatic Malignant Neoplasm in the Bone, Metastatic Malignant Neoplasm in the Soft Tissues, Metastatic Prostate Carcinoma, Prostate Adenocarcinoma, Recurrent Prostate Carcinoma, Stage IV Prostate Cancer	pTVG-HP: DNA encoding prostatic acid phosphatase (PAP)	Pembrolizumab	pTVG-HP: ID Pembrolizumab: IV	University of Wisconsin, Madison
**NCT01322802**	Phase I	Completed			Stage III Ovarian Epithelial, Cancer, Stage III Ovarian Germ Cell Tumor, Stage IV Ovarian Epithelial Cancer, Stage IV Ovarian Germ Cell Tumor	pUMVC3-hIGFBP-2 encoding human Insulin-Like Growth Factor-Binding Protein 2 (hIGFBP-2)	–	ID	National Cancer Institute (NCI), University of Washington
**NCT00034554**	Phase I	Completed			Stage III and IV Melanoma	DNA encoding gp75	–	IM	Memorial Sloan Kettering Cancer Center
**NCT00398073**	Phase I	Completed			Intraocular melanoma, cutaneous melanoma	DNA encoding mouse gp100	–	IM or particle-mediated epidermal delivery (PMED)	Memorial Sloan Kettering Cancer Center
**NCT00561756**	Phase I	Completed			Recurrent B-cell Lymphoma	DNA encoding murine extracellular domain of CD20	–	IM	Memorial Sloan Kettering Cancer Center
**NCT00788164**	Phase I	Completed			HPV16+ Cervical Intraepithelial Neoplasia 3 (CIN3)	DNA vaccine encoding HPV16 E7 linked to Mycobacterium tuberculosis heat shock protein 70 (HSP70) and signal peptide (pNGVL4a-Sig/E7(detox)/HSP70) and TA-HPV	Topical Imiquimod	IM	Johns Hopkins
**NCT03532217**	Phase I	Completed			Metastatic Hormone-Sensitive Prostate Cancer	DNA vaccine encoding neo-antigens	PROSTVAC, Ipilimumab, Nivolumab	IM with electroporation	Washington University School of Medicine
**NCT00807781**	Phase I	Completed			Metastatic Breast Cancer	DNA encoding mammaglobin-A	–	IM using a jet delivery device	Washington University School of Medicine
**NCT03655756**	Early Phase I	Completed			Unresectable stage III or IV cutaneous melanoma	DNA encoding Emm55 Streptococcal Antigen	–	Intratumoral	Morphogenesis, Inc.
**NCT00988559**	Phase I	Completed			HPV16+ Cervical Intraepithelial Neoplasia (CIN2/3)	DNA vaccine encoding HPV16 E7 linked to calreticulin (pNGVL4a-CRT/E7(detox))	Imiquimod	IM, particle-mediated epidermal delivery (PMED), or intralesional	Johns Hopkins
**NCT01634503**	Phase I	Completed			HPV16 or HPV18-associated Cervical intraepithelial neoplasia 3 (CIN3)	GX-188E: DNA encoding HPV-16 and HPV-18 E6 and E7	–	IM with electroporation	Genexine, Inc.
**NCT00580060**	Early Phase I	Completed			Melanoma	DNA encoding human GM-CSF as an adjuvant for peptide vaccines encoding gp100 and tyrosinase	–	Subcutaneous	Memorial Sloan Kettering Cancer Center
**NCT00033228**	Phase I/II	Completed			Stage IV cutaneous melanoma	DNA encoding Melan-A and tyrosinase	–	Intranodal	Mannkind Corporation
**NCT00698100**	Phase I	Completed			Cutaneous melanoma	Arm I: DNA encoding human tyrosinase Arm II: DNA encoding murine tyrosinase	–	IM	Memorial Sloan Kettering Cancer Center
**NCT00096629**	Phase I	Completed			Renal cell carcinoma	Arm I: DNA encoding human PSMA Arm II: DNA encoding murine PMSA	–	IM	Memorial Sloan Kettering Cancer Center
**NCT00471133**	Phase I	Completed			Intraocular and cutaneous melanoma	DNA encoding murine tyrosinase	–	IM with electroporation	Ichor Medical Systems Incorporated
**NCT01304524**	Phase II	Completed			HPV16 or HPV18-associated Cervical Intraepithelial Neoplasia Grade 2/​3 (CIN2/3)	DNA encoding HPV-16 and HPV-18 E6 and E7		IM with electroporation	Inovio Pharmaceuticals
**NCT00381173**	Phase I	Completed			Breast Cancer, Ovarian Cancer, Prostate Cancer, Colon Cancer, Renal Cancer	DNA encoding CYP1B1 formulated within biodegradable microencapsulated particles	Cyclophosphamide	ZYC300: IM Cyclophosphamide: IV	Eisai Inc.
**NCT01094405**	Phase II	Completed			EBV-associated nasopharyngeal carcinoma	A recombinant modified vaccinia Ankara (MVA) viral vector encoding the EBV viral tumor antigens EB nuclear antigen 1 (EBNA1) and latent membrane protein 2 (LMP2)	–	–	Chinese University of Hong Kong
**NCT02411786**	Phase I	Completed			Metastatic Prostate Cancer	DNA encoding the ligand-binding domain of the human androgen receptor	With or without Rhesus GM-CSF	ID	University of Wisconsin, Madison
**NCT01440816**	Phase II	Completed			Merkel Cell Carcinoma	DNA encoding IL-12 gene	–	Intratumoral with electroporation	OncoSec Medical Incorporated
**NCT00929526**	Phase III	Completed			HPV16 and HPV18-associated Cervical Intraepithelial Neoplasia	–	–	–	GlaxoSmithKline
**NCT00023647**	Phase I	Completed	Stage IV Melanoma			DNA encoding two epitopes of tyrosinase	–	intranodal	Mannkind Corporation
**NCT00582140**	Phase I	Completed	Prostate cancer			DNA encoding human PAP	rhGM-CSF	ID	University of Wisconsin, Madison
**NCT02172911**	Phase I/II	Completed	HPV16 or HPV18-associated cervical carcinoma			DNA encoding HPV16 and HPV18 E6/E7 antigens	–	IM with electroporation	Inovio Pharmaceuticals
**NCT00455221**	Phase I/II	Completed	CML			DNA encoding IL-12 and GM-CSF in separate plasmids as adjuvant for poly-epitopic peptide vaccine	Imatinib, BCR-ABL multi-epitope peptide vaccine	Subcutaneously	Tehran University of Medical Sciences
**NCT00019448**	Phase II	Completed	Stage IV Melanoma			DNA encoding gp100	With or without IL-2	DNA vaccine: IM or ID IL-2: IV	National Cancer Institute (NCI)
**NCT01486329**	Phase I	Completed	Stage IV Pancreatic Cancer			VXM01 based on live, attenuated Salmonella bacteria, containing a plasmid encoding VEGFR-2	–	Drink solution	Vaximm GmbH
**NCT01341652**	Phase II	Completed	Non-metastatic prostate cancer			rhGM-CSF with or without pTVG-HP (encoding PAP)	–	Intradermal	University of Wisconsin, Madison
**NCT00423254**	Phase I	Completed	Advanced solid malignancies (ovarian, Melanoma, Renal, Prostate and more)			DNA encoding PRAME and PSMA (pPRA-PSM)	Peptide vaccine including E-PRA and E-PSM derived from PRAME and PSMA, respectively	Intranodal	Mannkind Corporation
**NCT05422781**	Phase I	Completed	Polyomavirus-associated Merkel Cell Carcinoma			DNA encoding a mutated form of the large T antigen (LT) of Merkel cell polyomavirus (MCPγV) fused to LAMP-1	–	IM	Immunomic Therapeutics, Inc.
**NCT00450463**	Phase II	Completed	Prostate cancer			DNA encoding human GM-CSF and PSA in a viral vector	PROSTVAC-F/TRICOM Flutamide	–	National Cancer Institute (NCI)
**NCT02718443**	Phase I	Completed	Glioblastoma			VXM01 based on live, attenuated Salmonella bacteria, containing a plasmid encoding VEGFR-2	–	Drink solution	Vaximm GmbH
**NCT04160065**	Phase I	Active, not recruiting	Merkel Cell Carcinoma, Cutaneous Squamous Cell Carcinoma, Non-Melanoma Skin Cancers			DNA encoding Emm55, an immunogenic bacterial protein (IFx-Hu2)	–	Intralesional	TuHURA Biosciences, Inc.
**NCT05242965**	Phase II	Recruiting	stage IV non-small cell lung cancer			DNA encoding CD105/Yb-1/SOX2/CDH3/MDM2 (STEMVAC)	GM-CSF (Sargramostim)	IV	University of Washington
**NCT04329065**	Phase II	Recruiting	Breast cancer			DNA encoding HER-2, IGF-1R, and IGFBP-2 (WOKVAC vaccine)	Paclitaxel, Trastuzumab, Pertuzumab	ID	University of Washington
**NCT04079166**	Phase II	Recruiting	Malignant melanoma			DNA encoding an antibody framework (ImmunoBody^®^) expressing cytotoxic T-cell epitopes derived from Trp-2 and gp100	Nivolumab, Ipilimumab, pembrolizumab	IM	Scancell Ltd
**NCT05286060**	Phase II	Recruiting	Head and Neck Squamous Cell Carcinoma			GX-188E: DNA encoding HPV16/18 E6 and E7, a tissue plasminogen activator signal sequence, and an FMS-like tyrosine kinase 3 ligand	Pembrolizumab GX-17 (IL-7 as a T-cell growth factor)	IM with electroporation	Yonsei University
**NCT05455658**	Phase II	Recruiting	Triple-negative breast cancer			DNA encoding CD105/Yb-1/SOX2/CDH3/MDM2 (STEMVAC)	GM-CSF (Sargramostim)	IV	University of Washington
**NCT02529930**	Phase I/II	Completed	High-Grade Cervical Intraepithelial Neoplasia			VB10.16: DNA encoding a mutation-inactivated recombinant protein of HPV-16 E6 and E7, and human chemokine ligand3-like1 (CCL3L1)	–	IM	Nykode Therapeutics ASA
**NCT05799144**	Phase II	Recruiting	Oropharyngeal Carcinoma			pB1-11: DNA encoding HPV16/18 E6/E7 and HSP70	Pembrolizumab TA-HPV vaccine: a live recombinant vaccinia virus expressing HPV16/17 E6/E7	IM	Michael K. Gibson
**NCT02780401**	Phase I	Active, not recruiting	HER2-Negative Breast Cancer			DNA encoding HER-2, IGF-1R, and IGFBP-2 (WOKVAC vaccine)	GM-CSF (Sargramostim)	ID	University of Washington
**NCT05354323**	Phase I	Recruiting	Adult solid tumors			NECVAX-NEO1: personalized Ty21a-based vaccine encoding neoantigens	anti-PD-1 or anti-PD-L1 monoclonal antibody checkpoint inhibitor monotherapy	Drink solution	NEC OncoImmunity AS
**NCT05743595**	Phase I	Recruiting	Unmethylated Glioblastoma			DNA encoding personalized neoantigens	Retifanlimab	IM with electroporation	Washington University School of Medicine
**NCT03603808**	Phase II	Active, not recruiting	HIV-Positive High-Grade Anal Lesions			VGX-3100: DNA encoding HPV16/18 E6/E7	–	IM with electroporation	AIDS Malignancy Consortium
**NCT04397003**	Phase II	Recruiting	Extensive-stage Small Cell Lung Cancer			DNA vaccine encoding neo-antigens	Durvalumab	IM with electroporation	Washington University School of Medicine
**NCT04989946**	Phase I/II	Recruiting	Prostate cancer			pTVG-AR: DNA encoding androgen receptor ligand-binding domain	Degarelix, Nivolumab, Cemiplimab, Fianlimab	ID	University of Wisconsin, Madison
**NCT03600350**	Phase II	Active, not recruiting	Prostate cancer			pTVG-HP: DNA encoding Prostatic Acid Phosphatase (PAP)	Nivolumab GM-CSF	ID	University of Wisconsin, Madison
**NCT04131413**	Phase I	Recruiting	HPV16 Positive Cervical Neoplasia			pNGVL4aCRTE6E7L2: DNA encoding HPV-associated antigens	–	IM with electroporation	Sidney Kimmel Comprehensive Cancer Center at Johns Hopkins
**NCT04090528**	Phase II	Recruiting	Castration-Resistant, Metastatic Prostate Cancer			pTVG-HP: DNA encoding human prostatic acid phosphatase (PAP) or pTVG-AR: DNA encoding androgen receptor ligand-binding domain	Pembrolizumab	ID	University of Wisconsin, Madison
**NCT00436254**	Phase I	Active, not recruiting	Her-2 Positive Stage III-IV Breast Cancer or Ovarian Cancer			pNGVL3-hICD: DNA plasmid encoding the intracellular domain (ICD) of the HER-2/neu proto-oncogene	GM-CSF (Sargramostim)	ID	University of Washington
**NCT04015700**	Phase I	Active, not recruiting	Unmethylated Glioblastoma			DNA encoding tumor neoantigens	DNA plasmid encoding IL-12	Not specified	Washington University School of Medicine
**NCT02204098**	Phase I	Active, not recruiting	Breast cancer			DNA encoding Mammaglobin-A	Neoadjuvant endocrine therapy or chemotherapy	Not specified	Washington University School of Medicine
**NCT01209871**	Phase I	Active, not recruiting	Lymphoplasmacytic Lymphoma			DNA encoding macrophage inflammatory protein 3 alpha (MIP3a)-fused lymphoma idiotype	–	ID	M.D. Anderson Cancer Center
**NCT03750071**	Phase I/II	Active, not recruiting	Recurrent Glioblastoma			VXM01: Ty21a transformed with a plasmid encoding VEGFR-2	Avelumab	Not specified	Vaximm GmbH
**NCT06344715**	Phase I	Recruiting	Metastatic Castration-resistant Prostate Cancer			SL-T10: DNA encoding cancer-specific neoantigens	Pembrolizumab GX-17: a T-cell growth factor	IM	SL VAXiGEN
**NCT05698199**	Phase I	Active, not recruiting	Glioblastoma			ITI-1001: DNA encoding three CMV antigens (pp65, gB, IE-1) and LAMP-1	–	IM	Immunomic Therapeutics, Inc.
**NCT06088459**	Phase I	Recruiting	Hepatocellular Carcinoma			DNA vaccine (NWRD06) following radical resection	–	IM with electroporation	Newish Technology (Beijing) Co., Ltd.
**NCT03988283**	Phase I	Not yet recruiting	Pediatric Recurrent Brain Tumor			DNA vaccine encoding tumor neoantigens	–	IM with electroporation	Washington University School of Medicine
**NCT03439085**	Phase II	Active, not recruiting	Recurrent or Metastatic Human Papillomavirus-associated cancers			DNA plasmid encoding IL-12 and HPV antigens	Durvalumab	IM with electroporation	M.D. Anderson Cancer Center
**NCT02157051**	Phase I	Not yet recruiting	HER2-Negative Stage III-IV Breast Cancer			DNA encoding CD105/Yb-1/SOX2/CDH3/MDM2 (STEMVAC)	Recombinant human GM-CSF (rhGM-CSF)	ID	University of Washington
**NCT06276101**	Phase I	Recruiting	High-grade Squamous Intraepithelial Lesion (HSIL)			NWRD08: DNA encoding HPV-16 and HPV-18 antigens		IM with electroporation	Newish Technology (Beijing) Co., Ltd.

Immune-stimulatory cytokines can also tag along in activating the immune system. They can be encoded in the same plasmid encoding the tumor antigen(s), in a separate plasmid, or injected as adjuvants. The most commonly used cytokines are IL-2, IL-12, and GM-CSF (18).

IL-2 is one of the most commonly used cytokines in combination with cancer vaccines. It induces the activation of T-cells and their differentiation into both effector and regulatory T-cells. FDA approved its application in patients with metastatic melanoma and renal cell carcinoma (RCC) since multiple trials demonstrated its safety and efficacy in limiting tumor progression. In a preclinical study, BALB/c mice were injected with plasmids encoding CML antigen (BCR-ABL) with or without IL-2. It was demonstrated that mice injected with plasmids encoding BCR-ABL and IL-2 developed a stronger CD8+ and humoral immune response. The serum levels of IFN-γ were also higher in this group (97). In a pilot clinical study, 8 patients with HER2/neu positive metastatic breast carcinoma were treated with a DNA vaccine encoding full-length signaling-deficient HER2 combined with low dose IL-2, GM-CSF, and trastuzumab (Herceptin). The vaccine was shown to be safe with no severe side effects. A significant CD8+ T-cell response and endogenous anti-HER2 humoral response were detected. In a subgroup of patients, the humoral response could be detected several years after the administration of the last dose of the vaccine (98). In another clinical trial, a DNA vaccine encoding gp100 was administered in patients with stage IV melanoma with or without intradermal injection of IL-2, the results of which have been equivocal (58).

IL-12 is another pro-inflammatory cytokine that has improved the clinical efficacy of some conventional cancer therapeutics. In a clinical trial, 15 patients with Merkel cell carcinoma (an aggressive type of virus-associated cutaneous cancer) were injected with plasmid DNA encoding IL-12 followed by electroporation. The vaccine was injected into cutaneous, subcutaneous, or nodal lesions since the systemic administration of IL-12 induces severe side effects. Three patients had localized disease, all of whom went through definitive surgery and/or radiotherapy with a considerable progression-free survival (PFS) and one of them showed a complete pathologic response. In the other 12 patients with metastatic disease, the vaccine induced three partial responses and one stable disease, while eight patients had progressive disease. Tumor biopsy indicated IL-12 and TNF-α production in the TME. Regression of both injected and non-injected lesions was observed (58).

In a phase I clinical trial in the recruiting stage, SL-T10, a DNA vaccine encoding multiple cancer neoantigens is administered to 78 patients with metastatic castration-resistant prostate cancer (mCRPC), along with GX-17 (IL-17) and intravenous pembrolizumab. The primary objective is to evaluate treatment-emergent and serious adverse events and the secondary objective is to assess the immunological, clinical, and molecular (PSA level) response of the patients in this mode of treatment (NCT06344715).

GM-CSF is widely used in clinical trials regarding cancer vaccines because of its effect on T-cell activation and proliferation and APC maturation, although there is evidence that it may promote myeloid-derived suppressor cells (MDSC) recruitment into the TME, tampering with the anti-tumor immune response (18). GVAX is a cell-based cancer vaccine composed of tumor cells transfected with DNA encoding GM-CSF. It can be either autologous or allogeneic. Several studies have demonstrated the vaccine’s efficiency in inducing a potent immune response, tumor regression, and survival prolongation (99, 100). In another clinical trial, 18 patients with metastatic castration-resistant prostate cancer were treated with Sipuleucel-T [a DC-based cancer vaccine targeting prostatic acid phosphatase (PAP)] alone or combined with intradermal co-administration of a DNA vaccine encoding PAP and GM-CSF. There were no notable differences between the two arms regarding T-cell immunity but the patients receiving booster DNA vaccine and GM-CSF developed a much stronger antibody-mediated immune response. Progression-free survival (PFS) was nearly the same between both study groups. Overall the booster vaccine and GM-CSF induced an augmentation and diversification of the immune response (101). In a phase II clinical trial, 98 patients with newly diagnosed homologous recombinant-proficient advanced ovarian cancer will receive either AST-201 [a DNA vaccine encoding insulin-like growth factor binding protein-2 (IGFBP-2)] combined with recombinant human GM-CSF (rhGM-CSF) and chemotherapy (paclitaxel and carboplatin) or placebo combined with GM-CSF and chemotherapy. The vaccine will be injected in 3 shots intradermally. The patients have already undergone tumor debulking before the treatment. The primary objective is to determine the progression-free survival in each arm and compare them to elucidate the efficiency of the combination of the vaccine with GM-CSF and chemotherapy, while the secondary objective is to investigate the adverse events and immunological responses. (NCT05794659).

Other immune-stimulatory cytokines including IL-7, IL-15, and IFN-γ have also been successfully combined with DNA vaccines (102, 103).

As described earlier bacterial plasmids can stimulate the innate immune response and promote a pro-inflammatory TME through toll-like receptors (TLRs) especially when the DNA sequence is rich in unmethylated CpG islands. To further exploit this phenomenon, several DNA vaccine enhancement strategies rely on the simultaneous introduction of TLR agonists or TLR adaptive molecules.

Since adding TLR agonists can result in dangerous side effects including hemodynamic shock and exacerbation of autoimmune conditions, the attention was spanned towards TLR adaptive molecules which are safer. In a preclinical study, the effect of combining TLR adaptive molecules with DNA vaccines was evaluated in BALB/c mice. DNA vaccines targeting Influenza HA antigen or E7 (on mouse lung carcinoma) were co-administered with plasmids encoding a TLR adaptive molecule, either toll-interleukin-1 receptor domain-containing adaptor-inducing beta interferon (TRIF) or myeloid differentiation factor 88 (MyD88). It was demonstrated that adding MyD88-encoding plasmid to the treatment regimen preferentially augments the humoral immune response while adding TRIF-encoding plasmid mainly activated the cell-based immune response. The upregulation of pro-inflammatory cytokines including IFN-γ, TNF-α, and Th1-promoting cytokines (including IL-12 and IL-18) was also evident.

Several studies have demonstrated that CD4+ T-helper cells are crucial for the induction of a potent cytotoxic lymphocytic immune response. Based on this evidence, T-helper (Th) peptides are being used in combination with DNA vaccines to potentiate the resulting immune response by facilitating helper T-cell activation (104–110). As previously discussed, APCs use class II MHC molecules to present antigenic peptides to T-lymphocytes. An essential component of the MHC II-mediated antigen presentation is the invariant chain which binds with the peptide binding groove to prevent premature antigen loading and is later replaced with an antigenic peptide. The domain that binds the peptide groove is called CLIP (class II-associated Ii peptide). By replacing the CLIP region with a peptide called Pan DR-binding epitope (PADRE), this molecule can be inserted into the peptide-binding groove, but this time the PADRE peptide can’t be replaced with antigens and stays there. Then the PADRE peptide will be presented to the CD4+ T-cells which will activate them and provoke them to secrete cytokines that are necessary for CTL activation. This concept was used in a preclinical trial in which a DNA molecule encoding the invariant chain (in which the CLIP sequence was replaced with PADRE), was co-administered with a DNA vaccine encoding a single chain trimer (SCT) containing an immune-dominant CTL epitope of HPV-16 E6 antigen (SCT-E6 DNA). The E6-specific CD8+ T-cell immune response was far stronger using both of these DNA molecules compared to using only SCT-E6 DNA (109, 111–114).

The main challenge in DNA vaccine therapy is the circumvention of central and peripheral tolerance. By applying ICB one can release the breaks on the immune system. Inhibitory receptors including CTLA-4, PD-1, TIM-3, and LAG-3 can be targeted in ICB therapy (45). Especially in tumors with a higher mutation burden, ICB therapy is more likely to induce an effective immune response (115, 116). This is probably due to the fact that tumors with a high mutation burden have more neoantigens that could be detected by the immune system, so the activation of the immune system using checkpoint blockers promotes a broader and stronger immune response (65). In one clinical trial, patients with metastatic castration-resistant prostate cancer were treated with a DNA vaccine encoding prostatic acid phosphatase (pTVG-HP) combined with pembrolizumab. The vaccine was administered intradermally and the ICB treatment was administered intravenously. One grade IV adverse event (hyperglycemia) occurred and 42 percent of patients developed an immune-related adverse event of grade 2 or higher. No complete clinical response was observed but a partial clinical response was recorded in one patient who was later found to have a tumor with a high degree of microsatellite instability. Thirty-two percent of patients had a progression-free survival of greater than 6 months. Immune response to PAP was detected in 20 percent of patients, using IFN-γ and granzyme-B fluorescent ELISpot. Cytokines associated with CD8+ T-cell activation were elevated in almost all of the subjects’ serum (101).

Chemotherapy has been a cornerstone of cancer treatment for a long time. In theory, chemotherapy not only directly kills cancer cells, but it also causes the release and spread of the tumor antigens and the removal of the immunosuppressive cells. Because of this and the prominent role of chemotherapy in cancer management, some studies have combined DNA vaccines with chemotherapy to assess the immunologic and clinical responses evoked. In a phase I clinical trial, ZYC300, a DNA vaccine encoding the carcinogen activator cytochrome P450 1B1 (CYP1B1) was combined with intravenous administration of cyclophosphamide. CYP1B1 is known as a universal cancer antigen as it is expressed in almost all human cancer types but is rarely expressed in normal tissues. The vaccine contains a plasmid DNA encapsulated in biodegradable poly-DL-lactide-coglycolide microparticles and it is administered intramuscularly every two weeks for a maximum of 12 cycles. Seventeen patients with different types of progressive advanced-stage cancer (including breast, prostate, colon, and renal cancer) were treated with the vaccine and chemotherapy. No significant adverse events were observed. In an ELISpot assay, 6/17 patients developed an anti-CYP1B1 T-cell immune response. The responders had received fewer prior regimens and more vaccination cycles compared to immunologic non-responders. Two restagings were carried out, one after receiving 6 cycles and one after the completion of 12 cycles of vaccination. Five patients developed a stable disease (SD) following 6 cycles of vaccination, two of whom retained the stable disease after the completion of the 12 cycles, and one of whom remained stable 16 months after the last dose of vaccination. All of the other subjects progressed eventually. Out of the eleven patients who did not respond, three passed before receiving the salvage therapy, seven progressed through the next salvage therapy, and one developed a complete remission after surgical removal of the tumor and administration of an experimental autologous cancer vaccine. Curiously, all five immune-responders who developed progressive disease following vaccination had a clinically significant response to salvage treatment. This suggests an association between the induction of an immune response against cancer antigens encoded in the vaccine and the response to the salvage treatment (117). In a preclinical study, the safety and efficacy of a prime-boost vaccine combined with low-dose cyclophosphamide was evaluated in a murine breast cancer model (4T-1) of BALB/c mice. The vaccine encoded Fibroblast Activation Protein α (FAPα) which is an antigen expressed in cancer-associated fibroblasts (CAFs), but not usually expressed in normal differentiated cells. The CAFs are responsible for creating a harsh immune-inhibitory TME by recruiting immunosuppressive cells and secreting cytokines such as IL-10, TGF-β, and CXCL12. Targeting FAPα will potentially help the immune system overcome these barriers and develop a strong anti-tumor response (118–128). Apart from being a potent chemotherapy agent, cyclophosphamide has been shown to modulate immune-suppressive TME when administered in low doses by inhibiting regulatory T-cells and down-regulating IL-10. In this way, chemotherapy can have a synergistic effect with the cancer vaccine in developing a strong immune response which is necessary in the battle with cancer (129–131). On the other hand, a DNA-prime MVA-boost strategy is regarded as a more efficacious mode of vaccine delivery than using DNA vaccine or MVA vaccine alone, as it can induce robust CD8+ and CD4+ immune responses (132, 133). To determine the most efficacious mode of delivery, mice were sorted into four cohorts, each receiving either phosphate-buffered saline (PBS), a DNA vaccine encoding FAPα (CpVR-FAP) alone, the viral vector vaccine (MVA-FAP) alone, or receiving both the DNA vaccine and the viral vector vaccine in a prime-boost fashion. It was determined that the prime-boost vaccination induced more cytotoxic lymphocytes and antigen-specific IFN-γ secreting T-cells in an ELISpot assay. In the next phase, mice were sorted into four cohorts again, but this time they received either PBS, cyclophosphamide, DNA+MVA, or DNA+MV+cyclophosphamide. It was determined that administering all three treatments at the same time will induce a greater tumor inhibition rate, a longer survival rate, a more potent CD8+ immune response, and an increase in the expression of IFN-γ and IL-β. Also, there was a bigger reduction in the expression of IL-10, FAPα, and other CAF-related factors (including but not limited to SDF-1 and HOF). There was also a marked reduction in the number of regulatory T-cells in tumor specimens. Overall, this preclinical study revealed a significant synergistic effect of adding cyclophosphamide to a prime-boost anti-cancer vaccination schedule which merits further evaluation on human malignancies (134). In a phase II clinical trial in the recruiting stage, 16 patients with HER2-positive breast cancer of different stages will receive a DNA vaccine encoding IGFBP2, HER2, and IGF1R together with paclitaxel and HER2-targeted monoclonal antibody treatment as neoadjuvant therapy before the surgical removal of the tumor. The vaccine is given in 3 doses in the absence of disease progression and severe toxicity. The patients are projected to be followed annually for up to 5 years after the completion of the vaccination. NCT04329065.

Another approach is to combine DNA vaccines with radiotherapy. In one clinical trial, a DNA vaccine encoding HPV-16/18 E7 and E6 was co-injected with a plasmid DNA encoding IL-12 in ten patients with biopsy-proven stage IB-IVB HPV16/18 positive cervical cancer. Two to four weeks before vaccination, all patients went through chemo-radiotherapy using external beam radiotherapy (EBRT) and intracavitary techniques (interstitial brachytherapy). The vaccine was injected intramuscularly followed by electroporation. Overall, 8/10 patients had humoral or cellular immune responses in ELISpot and ELISA assays, 6/10 had *de-novo* sero-response against HPV-16 antigens and 6/10 had *de-novo* sero-response against HPV-18 antigens. To assess local immune responses, cervical biopsies were taken before chemoradiation (pre-CRT), after chemoradiation (post-CRT), and after the completion of vaccination (post-vaccination) using a multiplexed immunofluorescent assay. Compared to pre-vaccination samples, post-vaccination samples showed decreased epithelial cells (consistent with tumor regression) and decreased PD-L1^+^ CD8^+^, PD-1^+^ CD8^+^, and PD-L1^+^ CD68^+^ T-cells which are the usual suspects of creating a hostile immune-suppressive TME. Subjects were also assessed for the presence of HPV DNA in their bloodstream using HPV-PCR. Before chemo-radiotherapy, 6/10 patients were positive for HPV DNA, which was dropped to 2/10 patients after chemo-radiotherapy, and eventually, all patients were negative for HPV DNA following the completion of vaccination. The estimated progression-free survival was around 90 percent. Out of 8 evaluable subjects, 7 showed complete remission (CR), and one showed partial response (PR). PET/CT showed a decreased or stable hypermetabolic state of the tumor after vaccination (135).

Hormones play an important role in tumor metabolism and growth. Hormone deprivation is a mode of treatment in some types of cancer, most commonly prostate cancer. In a phase II clinical trial patients with non-metastatic castration-resistant prostate cancer (nmCRPC) were treated with flutamide (a non-steroidal androgen receptor antagonist) with or without PROSTVAC (a poxvirus-based DNA vaccine encoding PSA and three T-cell co-stimulatory molecules). Thirty-three subjects received flutamide alone (arm 1) and thirty-one subjects received flutamide and PROSTVAC (arm 2). The vaccine was well-tolerated with no significant adverse events. Seven patients in each arm had a PSA level reduction of more than 50 percent. The median time to treatment failure was 4.5 months for arm 1 and 6.9 months for arm 2. Although slightly improving the clinical response, the vaccine was unable to induce a significant immune response (136). In a phase I/II clinical trial in the recruiting phase, patients with newly diagnosed, high-risk prostate cancer will receive a DNA vaccine encoding the ligand-binding domain of the androgen receptor (pTVG-AR) combined with androgen deprivation therapy with Degarelix (a GnRH-receptor antagonist injected subcutaneously) and nivolumab/cemiplimab/fianlimab. The vaccine is injected intradermally. The patients are scheduled for prostatectomy following the treatment and the excised prostate tissue will be used for the evaluation of the pathological response rate. The primary outcomes measured in this trial are the pathological response rate, minimal residual disease, the incidence of adverse events, and the rate of toxicity (NCT04989946). Small molecules targeting growth receptors are another mode of treatment. The advent of imatinib marked a milestone in the treatment of chronic myeloid leukemia (CML) (18). These molecules function as blockers of the growth factors or their receptors, depriving tumor cells of these signals. In one trial, ten patients with chronic myeloid leukemia (CML) were treated with a poly-epitopic peptide vaccine and plasmids encoding IL-12 and GM-CSF as adjuvant. The patients were followed for seven years to evaluate the survival rate and long-term molecular response. The patients were previously and concomitantly treated with imatinib (A tyrosine-kinase inhibitor). All of the patients experienced cytogenetic improvement with 5 patients having complete cytogenetic response. Four patients developed major molecular response (MMR), while four other patients had significant reductions in BCR-ABL transcripts in their peripheral blood samples. The two non-responders developed complete molecular response (CMR) or MMR at some point in the seven-year follow-up period, signifying the endurance of the elicited immune response. Also, the serum levels of IFN-γ were elevated in most of the patients (137). Cancer vaccines can be used as adjuvant treatment following surgery. One example is the novel treatment of oral melanoma in dogs. Oral melanoma is the most common oral tumor in dogs. It is quite aggressive with metastases frequently detected at the time of diagnosis. Because of poor response to chemotherapy and radiotherapy, surgical removal is the treatment of choice for this malignancy but median survival time is no more than one year following surgery. To overcome this issue, cancer vaccines could be used as adjuvant treatment in these dogs following surgery. One study was conducted to evaluate the safety and efficacy of a DNA vaccine encoding human tyrosinase (hu-Tyr vaccine) as an adjuvant treatment for canine oral malignant melanoma (MM) after surgical removal of the primary tumor (the vaccine is considered a xenogeneic one). Fifty-eight dogs were prospectively enrolled and received four shots following a mean interval of 43 days after radical tumor excision, while fifty-three dogs were used as historical controls. No significant adverse events were observed. Time until death attributable to MM was markedly increased in those receiving the vaccine compared to historical controls, however median survival time could not be determined in the vaccinates since the majority of these subjects outlived the trials observation period (138, 139).

Oncolytic viruses are an emerging option for cancer immunotherapy. They are replication-competent antigen-agnostic viruses that selectively infect tumor cells. The immune-suppressive nature of the tumor micro-environment facilitates the growth of these viruses (140). Co-administering these viruses with DNA cancer vaccines might be a viable therapeutic option. However, we were not able to find a study incorporating both these treatments into its regimen.

## 7 Route of administration and boosting

One aspect of developing a cancer vaccine that is commonly overlooked is finding an optimal route of administration. As we know, there are several routes of administration for DNA vaccines, including intradermal, intramuscular, subcutaneous, intranodal, intratumoral, and intravenous. There is yet to be a consensus on which of them is more efficient as each of them has its pros and cons. Also, some techniques including electroporation, sonication, biolistic delivery, and DNA tattooing have been developed which aim at increasing the cellular uptake of the plasmid to increase the expression of antigens. In this section, we will discuss the pros and cons of the novel technologies regarding DNA vaccine delivery.

Intradermal delivery of vaccines has been used for centuries. It is popular partly because of the abundant resident APCs, particularly Langerhans cells which deliver the epitopes to the naïve T-cells of the draining lymphoid tissues. Trials have indicated that intradermal administration might be superior to intramuscular delivery because of the enhanced immune response and the lower required dose of the vaccine (141–144). One of the disadvantages of conventional intradermal administration is the use of needles which imposes the risk of needle-stick and cross-contamination. An innovative approach to address this problem is to deliver the antigens into the dermal or epidermal tissue using needle-free injectors. In this method a stream of high-pressure and high-velocity liquid penetrates the skin, supplying the Langerhans cells and other immune cells with the tumor antigens. It has several advantages over classic intradermal delivery including the elimination of needle-stick, reduced cross-contamination, and lower overall cost. It should be stated that there is a risk for cross-contamination through fluid suck-back and splash-back as observed by Weniger et al. (145). In one trial it was demonstrated that the intradermal injection using a needle-free apparatus called pyro-drive jet injector (PJI) confers a more intense protein expression (146–148). In a preclinical trial, C57BL/6 mice were injected with a DNA vaccine containing plasmids encoding ovalbumin (OVA) using a pyro-drive jet injector. Lymph node and spleen analysis showed a sizable expansion of OVA-specific CD8+ T-cells and CD4+ T-cells to a lesser extent. Also, there was increased transcription of IFN-γ and IL-4 genes. Tumor models expressing OVA were transplanted into the mice and a strong prophylactic and therapeutic response was observed among those receiving the vaccine. Humoral responses were also observed through the increased levels of IgG2a and IgG1 subclasses (146). Another approach to enhance antigen delivery in intradermal administration is the use of biolistic techniques, most notably the gene gun. The gene gun is a form of biolistic system that propels heavy metal particles, mostly gold micro-particles, covered with DNA or mRNA molecules. It can induce a potent immune response with a much lower dosage compared to intramuscular administration alone. Also, it has been demonstrated that the gene gun delivery induces a Th2-dominant immune response. T-helper2 cells are associated with a strong humoral immune response and the up-regulation of IFN-γ and IL-2. In a preclinical trial, a DNA vaccine encoding HPV-16 E7 protein linked to mycobacterium tuberculosis heat shock protein 70 (HSP70) was administered to C57BL/6 mice. The plasmid also contained two tandem repeats of CpG islands and a signaling peptide that enhances the immune response by mediating the secretion of the encoded antigens to be detected by the APCs. Three methods of administration were experimented in this trial: intramuscular, projector 2000, and helium-driven gene gun. It was demonstrated that administration via gene gun can generate more E7-specific IFN-γ+ CD8+ T-cells among splenocytes compared to the other two methods. A tumor model expressing E7 (TC-1) was injected subcutaneously to assess the therapeutic potential of the vaccine. The mice were euthanized and the lung weight and the number of pulmonary nodules were used for the evaluation of tumor response. The mice immunized with the gene gun delivery method had lighter lungs and fewer pulmonary nodules, although the statistical difference was not significant. Overall, using gene gun delivery, a more potent immune response was reached using a lower vaccine dosage (149).

Intramuscular delivery is another common route for the administration of vaccines, including DNA vaccines. Some of the advantages of this route include flexibility of vaccine dosage, the relative ease of administration, and less systemic and local side effects such as irritation, inflammation, necrosis, and granuloma formation (150). The muscle is rich in blood supply which improves the immune response and mitigates the local administration-site side effects. The most common site of vaccine administration is the deltoid muscle and anterolateral aspect of the thigh. Overall, the delivery routes in which the antigens are retained in the injection site for a longer time are associated with a more severe injection-site irritation. Because of this, routes in which the vaccine is mostly injected in adipose tissue such as subcutaneous injection and intramuscular injection into muscles covered with a thick layer of fat (like gluteal administration) are associated with a weaker induced immune response and a more intense local side effects. Also intramuscular injection of vaccines conjugated with salts (such as aluminum salts) which cause the retention of vaccine in the injection site for sustained release cause a more severe injection-site irritation (150). To improve the internalization of large DNA molecules into muscle cells, some physical augmentative methods have been developed. Electroporation (EP) is one of them. EP is an effective physical approach for the enhancement of nucleic acid uptake by APCs and other cells following DNA and mRNA vaccination. EP can be used following intratumoral, intradermal, and intramuscular administration (151). It works by transiently permeabilizing the cellular membrane and allowing the large nucleic acids to enter the cytosol. After cessation of the electrical field, the pores are closed and the nucleic acid is trapped inside the cell. There is also some evidence for the promotion of gene expression and secretion of pro-inflammatory cytokines following electroporation which will further improve the resulting immune response (151). A plasmid DNA encoding human PSA was injected intramuscularly into C57 BL/6 mice followed by electroporation. In a subgroup, the vaccine was co-administered with DNA oligonucleotides rich in CpG islands. The vaccine resulted in a significant anti-PSA humoral and cytotoxic immune response evaluated by ELISA and IFN assay. The vaccination considerably delayed the appearance of neoplastic lesions in mice following tumor challenge with TRAMPC1/hPSA (a prostate cancer cell line expressing PSA). Co-administration of the CpG-rich sequences furthered improved the immune responses and the prevention of tumor occurrence (152). It has been shown that electroporation might be superior to other physical methods including ultrasonication in inducing a potent immune response (153). Interestingly some trials have indicated that electroporation following booster vaccination alone might be superior to electroporation following both priming and boosting (154). DNA tattooing is another bizarre physical delivery system. In this approach, the plasmids are delivered via a permanent makeup device that is frequently used in cosmetic tattooing. By creating thousands of micro-punctures, the DNA molecules are delivered into keratinocytes and other skin cells. Also, this minor trauma activates the innate immune response, creating a hospitable environment for the development of a robust immune response, somewhat acting like an adjuvant. Although the expression of the antigen is transient, results have shown that the immune responses elicited by using a DNA tattooing are much stronger and it is developed in a much shorter interval than intradermal and intramuscular delivery, probably due to local injuries, wider surface area of application and an increased uptake of plasmids by resident APCs. This approach is safe but it is associated with skin irritation, swelling, and erythema. In a preclinical trial, C57BL/6 mice were treated with a plasmid DNA encoding L1 major capsid protein of HPV-16. The vaccine was delivered either intramuscularly with a simple injection or via tattooing. Pre-treatment with cardiotoxin or co-administration of a plasmid encoding murine GM-CSF was used as an adjuvant. The L1-specific cellular and humoral responses were significantly more potent than simple IM injection. Also, the co-administration of GM-CSF and pre-treatment with cardiotoxin noticeably enhanced the immune response following IM injection, but it did not meaningfully improve the L1-specific immune responses following DNA tattooing. Albeit, the immunity acquired by tattooing always surpassed that of the IM injection. Interestingly, the lymphocytes explanted from subjects treated with tattooing proliferated much more avidly compared to IM injection following stimulation with a non-specific mitogen. This may reflect the significant activation of the immune system and the release of a plethora of cytokines following tattooing, partly due to the local administration-site trauma and the release of danger signals. Overall, this trial demonstrated that choosing an appropriate route of administration might be more influential than using adjuvants. It should be noted that the process of DNA tattooing is rather tedious, and it might not be acceptable for some patients (155). Sonoporation and Laser beams are some other methods used to create temporary pores in the cellular membrane. Their safety and efficacy have been evaluated in a few trials (156–158). One of the unconventional routes of administration is the intratumoral (IT) route. During this process, the plasmid DNA encoding tumor antigen(s) is injected into the tumor. Sometimes the needle is guided to the tumor using ultrasonography or other imaging modalities. Compared to systemic administration routes, intratumoral injection elicits fewer adverse events which could disrupt the treatment. Also, the vaccine dosage required to obtain the same level of immune response is less compared to systemic administration which will make the vaccines more cost-effective. Using intratumoral injection of plasmid DNA encoding immune-stimulatory cytokines, one can break the immune-suppressive nature of the TME. In a preclinical trial, the intratumoral injection of DNA, encoding Endostatin and Angiostatin boosted the immune response elicited by a DNA vaccine and increased tumor-free survival in a melanoma mouse model (159). This can facilitate the anti-tumor immune response elicited by other anti-tumor treatments. In a phase II clinical trial, a DNA vaccine encoding IL-12 was administered to 15 patients, 4 of whom had objective tumor response (160). In a phase 1 clinical trial, patients with unresectable stage III or IV cutaneous melanoma were injected with IFx-Hu2.0 (a plasmid DNA encoding EM55, a streptococcal membrane protein) intralesionally. No grade 3 or higher adverse events were observed. Elevated T-cell infiltration was observed in post-vaccination lesion biopsy samples compared to the pre-vaccination ones. Also, higher IgG and IgM antibody levels against melanoma-associated antigens in the plasma were observed post-vaccination. Interestingly, in the non-injected lesions, higher levels of mRNA molecules encoding proteins associated with an innate immune response such as CXCL10, CXCL11, CXCL13, LAG3, and ICOS were observed compared to the pre-vaccination sample of the same lesions (161).

Intranodal administration of DNA vaccines might be unconventional and bizarre, but some researchers have shown that it might be quite efficacious. Lymph nodes are rich in APCs and lymphocytes. Direct injection of plasmids encoding antigens might create a stronger immune response, more rapidly. It also obviates the necessity for APC migration into lymph nodes. In a phase I clinical trial, patients with stage IV melanoma were treated with a DNA vaccine encoding a tyrosinase epitope. Two CpG islands were inserted into the DNA structure to enhance its immunogenicity. The patients were heavily pre-treated with chemotherapy and, IL-2. The vaccine was infused into a lateral superficial inguinal lymph node using ultrasonography. The duration of the infusion was 96 hours. The rationale behind this extended time of infusion was that the extended period of infusion might enhance the antigen uptake by APCs and boost the resulting immune response. The treatment was usually well-tolerated but 5 patients developed grade1/2 adverse events. 11/24 patients were deemed as immune responders according to a tetramer assay, but only 3 sustained this positivity in the follow-up period. 6/24 patients were positive for a delayed-type hypersensitivity test. The projected median overall survival for the patients was 15.2 months, compared to 7-9 months for stage IV melanoma from the start of other modes of treatment. A significant association between immune reactivity and clinical outcomes was noted in this trial. Out of 13 survivors at the end of the follow-up period, 9 were immune-responders, although the small sample size limits rigorous analysis of the data. Overall, this trial demonstrated the mild efficacy of the intranodal administration of DNA vaccines on metastatic melanoma but more importantly, it proved the practicality of repeated and extended infusion of DNA vaccines into lymph nodes (159).

The gastrointestinal system is an immune-competent system in humans with a large surface area. It has an extensive and complex immune system (including the Peyer’s patches) which could be exploited for cancer vaccination. Oral administration is a novel route for the delivery of cancer vaccines including DNA vaccines, which offers ease of administration and no local injection-site irritation as observed in other routes. Also, the activated T-cells following oral vaccination have a higher affinity for the tumors of the gastrointestinal system and could be used in many different GI tumors including gastric cancer, colorectal cancer, and pancreatic cancer. In a phase I clinical trial, an attenuated Salmonella Typhimurium transfected with VEGFR-2 encoding plasmid (VXM01) was administered orally in patients with locally advanced or stage IV pancreatic cancer. The vaccine was well-tolerated with no dose-limiting toxicity. Compared to the control group, a considerable rise in CTL-mediated anti-VEGFR2 immune response was noted among the vaccinated. Also, a prominent reduction of blood perfusion to the tumor was observed following vaccination, since VEGFR2 is an angiogenic mediator. Interestingly, a high percentage of patients had VEGFR2-specific T-cell responses before vaccination, which reflects a pre-existing spontaneous anti-angiogenic immune response without an external stimulus. Those with a higher level of anti-VEGFR2 immune response before vaccination had a more noticeable diminution of tumor blood flow. No significant difference between vaccinates and controls was observed regarding the clinical outcome. Only one vaccinated subject had a partial response with a significant drop in CA19.9 levels. This patient had developed a strong VEGFR2-specific immune response and a significant reduction of tumor perfusion (162).

Most of the vaccines we have require multiple administrations or ‘shots’ to effectively stimulate the immune system against a specific antigen. For instance, it is recommended for a person to receive a tetanus-diphtheria (Td) vaccine every 10 years following the prime vaccine (163). These multiple doses are essential for the majority of vaccines irrespective of the type of vaccine. Traditionally, the prime and booster vaccines are the same. However, research has indicated that using different types of vaccines encoding the same antigen might be superior to the conventional homologous prime-boost vaccination (164, 165). Intramuscular injection of DNA plasmids that encode TAA fused to the B subunit of Escherichia coli heat-labile toxin and the extracellular and transmembrane domain Her2, followed by boosting with an adenoviral vector vaccine carrying the same genes induced cell-based and humoral responses against both TAAs in mice. This mode of vaccination, induced therapeutic antitumor immunity against HER2+ mammary tumors and CEA+ colon tumors. On top of the immunogenicity of the vaccine, this trial demonstrated the safety and tolerability of the prime-boost vaccination (166). In another preclinical trial, a heterologous DNA prime-peptide boost vaccine schedule was evaluated on BALB/c mice with renal-cell carcinoma (RCC). RCC is an immunogenic tumor that responds poorly to chemotherapy and radiotherapy. However, immunotherapy is a viable option because of the tumor immunogenicity (167, 168). The DNA vaccine was composed of plasmid DNA encoding G250 protein (also known as carbonic anhydrase-9 that is abundantly expressed on RCC tumor cells but not on the normal non-cancerous cells) fused with the major immune-dominant region of the HBcAg gene. Some mice received plasmids encapsulated in polyethyleneimine (161) as the carrier to enhance the transfection efficiency. PEI can also have adjuvant effects to further augment the immune response. Compared to mice receiving the DNA vaccine or DNA-PEI alone, the group that received DNA-PEI + Peptide vaccine demonstrated significantly more potent cellular and humoral anti-G250 as evaluated by ELISA and ELISPOT assays (169). In a phase I clinical trial, 9 patients with stage III or IV HER2+ breast cancer were treated with a heterologous DNA prime-viral boost vaccine schedule. The plasmid was bicistronic encoding both the truncated epidermal growth factor receptor (HER2) and GM-CSF. The adenoviral vector contained the same HER2 sequence. Both vaccines were injected intramuscularly. The vaccines proved to be safe and well-tolerated. All 9 patients developed cell-based immune responses against HER2 but only 3 developed humoral responses. It should be noted that 5/9 patients received trastuzumab (an anti-HER2 monoclonal antibody) simultaneously. Because of this and the limited number of participants, the results of this trial could have been easily confounded (170). In a phase II clinical trial in the recruiting stage, 54 patients with HPV-positive oropharyngeal cancer will be vaccinated in a prime-boost fashion. pB1-11 is a DNA vaccine encoding HPV16/18 E6/E7 antigens linked to heat shock protein 70 (HSP70) which will be used as the priming vaccine. Booster vaccination will be conducted by the intramuscular administration of TA-HPV, an investigational recombinant vaccinia virus encoding the same antigens as the pB1-11. This vaccine platform is combined with pembrolizumab to further exploit the blockade of PD-1 on T-cells. The patients will be followed by computed tomography, magnetic resonance imaging (MRI), blood sampling, and tumor biopsy. NCT05799144.

## 8 Vectors

One of the major obstacles in the way of making an efficacious DNA vaccine against cancer is the low immunogenicity associated with DNA vaccines, which is at least partly due to the necessity of crossing multiple barriers including the cellular and nuclear membranes before the encoded antigens can be expressed (171). For a transgene to reach the nucleus, it has to enter the cell by pinocytosis or endocytosis, then it has to escape lysosomes, endosomes, and intracellular nucleases. Finally, it has to pass the nuclear membrane (171). All of these steps pose potential obstacles that diminish the immunogenicity of the vaccine. One solution to address this problem would be using nano-particle vectors or delivery vehicles. These vectors encapsulate or are otherwise covered with the payload (which is the DNA plasmid in this case) and offer several advantages over vaccination with a naked plasmid (172). Vectors protect the DNA molecules from degradation by several extracellular and intracellular nucleases, and other catalytic agents (173). They might also facilitate the selective facilitation of APCs, moderate toxicity issues, and enhance cellular and nuclear DNA uptake (174). Different types of these vectors have been designed and manufactured, some of the most notable ones will be discussed here.

### 8.1 Polymers

Polymers are large synthetic or natural molecules consisting of repetitive units called monomers. They are one of the most widely used materials to transport nucleic acids, chiefly because of their biosafety. One important perk of using polymers is the versatility of polymers. Each polymer has its own unique physical, chemical, and biological properties which will determine its application in different clinical settings. Other than that, each polymer can be tailored specifically to meet our requirements. These modifications can alter the preferred transfected type of cell, the specific type of immunity induced, and the rate of vaccine release from the injection site (175–177).

Chitosan is a widely used polymer for vaccination. It can be manufactured by treating chitin (a polysaccharide found in crustaceans) with an alkali. Regarding nucleic acid vaccines specifically, since chitosan assumes a positive charge, it can tightly bind to the nucleic acids which have an anionic nature through electrostatic forces (172). It is biocompatible with a very low incidence of toxicity (178, 179). It has inherent adjuvant effects as it has been proposed to stimulate type I Interferon release. This can augment the immune response through DC maturation and activation (180). It is highly insoluble with mucoadhesive properties that can justify its application in vaccination through mucosal routes (180). In a preclinical trial, a nano-chitosan-based delivery vehicle was used to deliver DNA plasmids encoding HPV-16 E7 into C57BL/6 mice. The vaccine was administered intramuscularly. Strong anti-E7 CD8+ and IFN-γ responses were observed. Also, significant anti-tumor responses were recorded in mice challenged with E7-expressing tumor cells (181). One strategy to improve vaccines made of chitosan nanoparticles is to functionalize these NPs by covering them with mannose moieties. That is because the surface of DC and other APCs is rich in mannose receptors, which can facilitate the internalization of nanoparticles if they are covered with mannose moieties (182). In one preclinical trial, an intranasal DNA vaccine encapsulated in mannosylated chitosan against tuberculosis showed promising results in mice with string IgA and cellular responses (183). Using molecules in the structure of the vector which will further improve transfection efficiency can help as well (184).

Poly (Lactic-co-glycolic acid) or PLGA for short is another widely used polymer for vaccination. It is biocompatible and biodegradable which led to its FDA approval (172, 185). Despite its safety, it has its shortcomings mostly associated with providing an optimal spatiotemporal payload release. The payload is released abruptly and fails to reach a homogenous release in the surrounding environment (186). It doesn’t offer the best protection of DNA from surrounding degradation agents either (172). All of these factors can preclude the formation of a robust immune response, capable of restricting tumor growth. To overcome these limitations, one approach would be combining PLGA with other materials with complementary properties to create composite nanoparticles that possess the advantages of both its constituents. These composite nanoparticles can provide enhanced release properties, more adamant DNA protection, and more formulation stability, as demonstrated in trials regarding Newcastle disease virus (NDV) and streptococcus agalactiae (187, 188).

Polyethylenimine (PEI) is another type of polymer frequently used for vaccination. The main advantage of this polymer is its versatility and structural flexibility. Based on the molecular weight and the degree of polymer branching, one can determine the transfection efficiency (189). The transfection efficiency and the rate of endosomal escape following internalization greatly increase with heavier, more branched polymers. This comes at a cost; the polymer becomes more cytotoxic. Conversely, the transfection efficiency and endosomal escape are challenged using low molecular weight, scarcely branched PEI polymers, but these polymers have a more favorable safety profile (190). So the main objective here is to find the fine line between transfection efficiency and toxicity. To attenuate the biosafety concerns regarding the application of high molecular weight, heavily branched PEI polymers, nanoparticles made from this polymer have been conjugated with polysaccharides, lipid molecules, and hydrophilic polymers that moderate the intense surface charge of heavy PEI polymers, which is responsible for their cytotoxicity (172, 191). Vaccines made from PEI polymers can be administered through novel, more unconventional routes such as intranasal (192) and oral (193) routes. In one preclinical trial, live attenuated bacteria were used for cancer vaccination in mice. These bacteria were coated with synthetic nano-particles comprised of cationic polymers and plasmid DNA encoding VEGFR2. The vaccine was delivered orally. The rationale for covering the bacteria with polymers was to protect the bacteria from stomach acid and to facilitate the endosomal escape following phagocytosis. This resulted in a wider dissemination of the bacteria in the body and a more intense immune response. Robust CD8+ and CD4+ T-cell activation and IFN-γ release were observed. Considerable suppression of tumor growth was observed which is the result of the inhibition of tumor angiogenesis (193). In one preclinical trial, BALB/c mice were treated with a transcutaneous injection of DNA vaccine encoding Trp2 (a melanoma-associated antigen) using micro-needles combined with mannosylated grafted cell-penetrating peptide-low molecular weight PEI copolymer (CPP-PEI1800-Man). Both of these treatment approaches enhance the transfection of skin-resident DC. Strong anti-tumor CD4+ and CD8+ T-cell responses were observed with enhanced secretion of IFN-γ and IL-12. The vaccination granted potent anti-tumor immunity so that the tumor growth was limited following the challenge with B16 tumor cells (194).

Poly (ethylene glycol) is commonly used for surface-functionalization and steric stabilization of other nanoparticles. It works by diminishing the surface charge of nanoparticles and preventing their interaction with serum proteins which can result in their premature clearance. The reticuloendothelial system (RES) has an important role in the clearance of nanoparticles; so using PEG polymers as a cover can help with the escape from clearance by the RES system (172). Although the application of PEG results in a more prolonged systemic circulation, it can hamper the transfection of APCs, rendering the immune response weak as a result (195, 196). To counteract this drawback, we can seek other ways to enhance APC maturation and transfection efficiency. One approach would be the incorporation of immune-stimulatory elements within the DNA structure such as CpG islands, TLR-9 agonists, and immune-stimulatory cytokines (197).

### 8.2 Lipids

Another class of non-viral nanoparticle vectors are lipid-based nanoparticles. These are synthetic sphere-shaped particles with at least one lipid bilayer, somewhat similar to that off the cellular membrane (198). These lipid-based particles have the upper hand regarding transfection efficiency and adjustability of surface properties (199). What keeps these vectors from vastly entering the clinical domain is their level of toxicity, instability in physiological conditions, and rapid clearance from the circulation (63). One type of these lipid-based nanoparticles are called liposomes. They are cationic nanoparticles made out of phospholipids and cholesterol. The complex of liposomes and nucleic acids encoding the desired antigen(s) is called a lipoplex. Similar to polymeric nanoparticles, lipid NPs can be surface-functionalized to facilitate target-specific delivery. For instance, liposomes have been covered with shikmic acid, a molecule similar to mannose which binds mannose receptors on the surface of DCs and help them to internalize liposomes (200).

Apart from their ability to carry antigen-encoding DNA into DC’ cytoplasm, liposomes can be used as vaccine adjuvants. For this, a liposome containing non-encoding DNA can be used as an adjuvant for another vaccine, regardless of the type of the vaccine. Research has indicated that this approach can boost the resulting immune response (201).

Another type of lipid-based nanoparticle delivery vehicles is called niosomes, which consist of cholesterol and non-ionic surfactants. In contrast to liposomes, they are not cationic and they form stable structures that somewhat compensate for the instability of liposomes *in vivo* (202, 203). To improve immunogenicity, niosomes have also been surface-functionalized with mannose moieties, which has interestingly improved the structure’s stability as well (204). In a preclinical trial, mice were injected with autologous DCs transfected with lipoplexes of a plasmid encoding melanoma-associated-antigens and liposomes of two lysinylated cationic amphiphiles with mannose-mimicking quinic and shikimic acid head-groups. More than 80 percent of the immunized mice experienced long-lasting protective immunity with considerable memory response (205).

### 8.3 Lipopolyplexes

These nanoparticles are made of antigen-encoding nucleic acids complexed with polymers and encapsulated in a lipid bilayer shell (172). In other words, this is a system that combines features of both polymeric and lipid-based nanoparticles. Consequently, it has the edges of both of these modalities, while the weaknesses of each modality are compensated for by the other modality (206, 207). Thus, these vaccines have high transfection efficiency, biodegradability, biocompatibility, *in-vivo* stability, circulation time, and less cytotoxicity in physiological conditions (206, 207). To further improve the stability and circulation time of nanoparticles and diminish their recognition by the immune system, the outer lipid layer is often designed with poly (ethylene glycol) moieties (172).

### 8.4 Virus-like particles

Virus-like particles (VLPs) are composed of self-assembling viral proteins expressed *in vitro* (208). They closely resemble viruses but they do not contain any viral genetic material (172), but they can be designed to have other genetic material within them, such as a DNA sequence encoding a tumor antigen (209, 210). The genetic material is usually incorporated using one of two approaches: in the first approach, the DNA molecule is pushed through the preformed VLP by submerging the VLP in a low ionic strength liquid containing the DNA molecule of interest. The VLP’s internal positive charge facilitates this transfer. In the second approach the VLPs are assembled in a liquid containing the DNA sequence of interest and again, the electrostatic interaction between the anionic DNA molecule and cationic VLP facilitates the process of incorporation (211). Unlike viral particles used in many vaccines, these VLPs do not impose any risk of insertional mutagenesis, reversion to virulence, and infection as seen in live attenuated viral vaccines (208). Other advantages include excellent adjuvant properties (212), morphological uniformity, biocompatibility, and ease of functionalization (213). One possible disadvantage of these methods is that the incorporated DNA molecule is usually small, usually up to 4kb (kilobases), but more novel approaches have been successful in the encapsulation of a 17kb long DNA sequence in a virus-like particle (211, 214).

### 8.5 Inorganic nanoparticles

Another class of nanoparticles are inorganic ones. These nanoparticles are usually made of metals such as gold, iron, and silver (172). These nanoparticles offer several advantages including ease of functionalization, biocompatibility, and well-known chemical properties (172). These inorganic NPs are inherently non-toxic and can be synthesized in a plethora of sizes, shapes, and aspect ratios (172).

Gold (Au) nanoparticles are commonly used as inorganic delivery vehicles for DNA vaccines. Some features have facilitated this utilization including tenability of surface chemistry, ease of production, and biocompatibility (172). In one preclinical trial, gold nanoparticles functionalized with a ligand containing shikimoyl and guanidinyl moieties (Au-SGSH) were used for the delivery of antigen-encoding plasmids into mice. Subcutaneous administration of near-infrared-labeled Au-SGSH demonstrated a significantly more intense accumulation of nanoparticles in the nearby lymph nodes compared to the non-targeting nanoparticles. One such nanoparticle, containing melanoma antigen recognized by T cells 1 (MART1)-encoding plasmid provoked an effective immune response against murine melanoma in the prophylactic setting. In a therapeutic setting, the same vaccine induced the limitation of tumor growth and prolongation of overall survival (215). Ferric (Fe) nanoparticles have garnered significant attention because of their low toxicity, affordability, and ease of surface-functionalization (172). Due to their magnetic properties, ferric NPs can be used in imaging, tumor ablation, and targeting a specific part of the body through an external magnetic field (216–218).

Other materials such as silver, layer double hydroxide, and calcium phosphate have also been used as vectors and adjuvants for DNA vaccines (213, 219–221).

## Conclusion

As our understanding of the pathophysiology of cancer expands, we have begun to appreciate the role of DNA as a valuable asset in cancer immunotherapy. Although several obstacles lie in the way of their development, DNA cancer vaccines are one of our most promising solutions for managing malignancies. Despite their current insufficiency to induce a clinically substantial immune response, these vaccines have shown their potential in numerous clinical trials and merit future investments. Several strategies are currently under investigation to fill in the gaps and form a more powerful and precise treatment. Optimizations regarding tumor antigen selection based on predicted immunogenicity, delivery vehicles, combination strategies, and routes of administration require further clinical research, and reviews like this can potentially broaden our view of the platform and provide a more comprehensive outlook on the opportunities of growth for DNA cancer vaccines. As we know, personalized medicine has already begun to force its way into modern medicine due to the abundance of evidence about its efficacy. In the past, we did not possess tools to analyze the inter-individual variability between different patients, so we prescribed roughly the same treatment for the same disease to different people. The emergence of next-generation sequencing and whole exome sequencing changed this narrative and made it possible to take individual markers into account before starting a treatment, one instance of which being the neo-antigen-encoding DNA vaccines. This has already shown its supremacy over conventional treatments. To further build up this supremacy, finding more markers to evaluate each patient is necessary. For example, understanding more about the type of immunosuppressive cells in the TME, the surface markers of tumor cells and cancer-associated fibroblasts (CAFs), and even serum tumor markers can help us specify our course of treatment to target the core of the problem. Each of these personal evaluations provides us with new drug targets to be used in combination with DNA cancer vaccines which can have additive or even synergistic effects. Discovering the markers will not only determine the content of our treatment but also its dosing, the vector, and the route of administration. This kind of personal treatment will not only improve clinical outcomes but will also alleviate the unpleasant side effects of the non-selective conventional treatments on the already in-pain cancer patient. This mode of personalized therapy may seem to be too expensive and non-cost-efficient to be used for each cancer patient, but it seems to be the only option we have to maximize the effectiveness of our treatment. Hopefully, in the near future, we will witness DNA cancer vaccines’ widespread application in clinical settings as a game-changing treatment.
